# Are there synergies from combining hygiene and sanitation promotion campaigns: Evidence from a large-scale cluster-randomized trial in rural Tanzania

**DOI:** 10.1371/journal.pone.0186228

**Published:** 2017-11-01

**Authors:** Bertha Briceño, Aidan Coville, Paul Gertler, Sebastian Martinez

**Affiliations:** 1 Knowledge and Learning Sector, Inter-American Development Bank, Washington, D.C., United States of America; 2 Development Impact Evaluation Unit, World Bank, Washington, D.C., United States of America; 3 Haas School of Business, University of California, Berkeley, California, United States of America; 4 Office of Strategic Planning and Development Effectiveness, Inter-American Development Bank, Washington, D.C., United States of America; London School of Hygiene and Tropical Medicine, UNITED KINGDOM

## Abstract

**Summary:**

The current evidence on handwashing and sanitation programs suggests limited impacts on health when at-scale interventions have been tested in isolation. However, no published experimental evidence currently exists that tests the interaction effects between sanitation and handwashing. We present the results of two large-scale, government-led handwashing and sanitation promotion campaigns in rural Tanzania, with the objective of tracing the causal chain from hygiene and sanitation promotion to changes in child health outcomes and specifically testing for potential interaction effects of combining handwashing and sanitation interventions.

**Methods:**

The study is a factorial cluster-randomized control trial where 181 rural wards from 10 districts in Tanzania were randomly assigned to receive sanitation promotion, handwashing promotion, both interventions together or neither (control). Interventions were rolled out from February 2009 to June 2011 and the endline survey was conducted from May to November 2012, approximately one year after program completion. The sample was composed of households with children under 5 years old in the two largest villages in each ward. Masking was not possible due to the nature of the intervention, but enumerators played no part in the intervention and were blinded to treatment status. The primary outcome of interest was 7-day diarrhea prevalence for children under five. Intermediate outcomes of behavior change including improved latrine construction, levels of open defecation and handwashing with soap were also analyzed. Secondary health outcomes included anemia, height-for-age and weight-for-age of children under 5. An intention-to-treat analysis was used to assess the relationship between the interventions and outcomes of interest.

**Findings:**

One year after the end of the program, ownership of improved latrines increased from 49.7% to 64.8% (95% CI 57.9%-71.7%) and regular open defecation decreased from 23.1% to 11.1% (95% CI 3.5%-18.7%) in sanitation promotion-only wards. Households in handwashing promotion-only wards showed marginal improvements in handwashing behavior related to food preparation but not at other critical junctures. There were no detectable interaction effects for the combined intervention. The associated cost-per-household gaining access to improved sanitation is estimated to be USD $194. Final effects on child health measured through diarrhea, anemia, stunting and wasting were absent in all treatment groups.

**Interpretation:**

Although statistically significant, the changes in intermediate outcomes achieved through each intervention in isolation were not large enough to generate meaningful health impacts. With no observable signs of interaction, the combined intervention produced similar results. The study highlights the importance of focusing on intermediate outcomes of take up and behavior change as a critical first step in large-scale programs before realizing the changes in health that sanitation and hygiene interventions aim to deliver.

**Trial registration:**

Clinicaltrials.gov NCT01465204

## Introduction

Understanding how to reduce enteric and diarrheal diseases has important implications for child morbidity, mortality and long-term growth. Diarrhea is the second largest killer of children under five [1], and poor nutrition at an early age can cause growth faltering and reduced cognitive development in the longer term [2]. Exposure to, and ingestion of, contaminated fecal matter is understood to be the main pathway through which children are exposed to diarrheal diseases and other afflictions that affect nutrient absorption and longer-term growth, such as soil-transmitted helminthes and environmental enteropathy [3]. Poor children in developing countries are most at risk of exposure.

Two of the most common development interventions to address this transmission mechanism are hygiene and sanitation promotion campaigns. The current evidence suggests limited effectiveness when at-scale interventions have been tested in isolation [4], [5], [6], [7], [8]. The lack of health impacts may be explained by modest changes in intermediate outcomes such as latrine coverage and use [9]. Since sanitation (and to a lesser extent handwashing) produce positive externalities, reaching threshold adherence levels may be critical to identifying health impacts [10]. In support of this hypothesis, a recent successful rural sanitation randomized control trial (RCT) finds relatively large improvements in coverage (30 percentage point increase) which translates into improvements in stunting outcomes and contrasts with the null effects found in other rural sanitation RCTs [11]. These changes in coverage are large relative to the current literature. A recent review of 27 interventions aimed at increasing sanitation coverage finds that coverage increases by 14 percentage points on average, while use increases by 10 percentage points [12]. While subsidies have been shown to be the most effective approach to increasing coverage levels [12],[13], even these often fall short of reaching levels where meaningful health effects may be detected. Similarly, for handwashing interventions, achieving sustained behavior change has remained elusive, despite convincing evidence that when behavior change does occur, diarrhea rates are significantly reduced [4], [5], [14].

Focusing on multiple transmission pathways simultaneously through a more comprehensive water, sanitation, and hygiene (WASH) package provides an alternative approach to improving health outcomes. Given the limitations described in [12], this may be more feasible than designing singular interventions that are more effective at increasing latrine coverage and sustaining improved handwashing practices. For instance, [15] finds large reductions in diarrheal incidence through a combined water and sanitation package delivered in India.

There is a strong rationale for believing that synergies between interventions may exist. Improved sanitation can reduce the presence of human fecal matter, but with poor hygiene practices children may continue to expose themselves to contaminants. A primary focus on containment of human excreta may also miss the important role of animal contamination. For instance, [16] find that animal fecal markers are more common than human markers in both public and household water sources. Conversely, improved hygiene may not be enough to shield children if poor sanitation increases the presence of fecal matter in homes and water sources. A natural question then arises–are there important complementarities between hygiene and sanitation interventions? If so, then testing each type of intervention in isolation may miss critical interaction effects that are larger than the sum of its parts.

This study represents the first cluster-randomized trial to explicitly test the interaction between handwashing and sanitation promotion. We measure the effects of two large-scale, government-implemented programs that promote handwashing with soap and improved sanitation in rural Tanzania to document the individual effects of each intervention conducted in isolation as well as interaction effects of implementing both interventions simultaneously. We hypothesized that the sanitation promotion intervention would increase sanitation coverage and use while the handwashing campaign would increase hygiene awareness and handwashing with soap at critical junctures. We anticipated that each intervention would improve health outcomes (diarrhea, anemia, weight-for-age and height-for-age) on their own, and would have a multiplicative effect when combined.

## Methods

### Ethics

Ethical clearance was received from the Western Institutional Review Board in December 2008 and complemented by local ethical clearance by the Tanzanian Institute for National Research in August 2011 in order to conduct the endline survey. As outlined in the approved IRB proposal, respondents agreed to participate after being read the approved informed consent form by the enumerator. This approach was used due to the low literacy prevalent in the study areas. The trial was registered as NCT01465204 at clinicaltrial.gov in November 2011 after the program interventions had begun, but before endline data had been collected. The original design of the study was led by economists and trial registration is relatively new to the development economics field even though it is standard practice in the health sciences. For instance, the American Economic Association trial registry only started in 2013. Following best practices in health sciences, the team decided to move forward with trial registration even though the intervention had already begun, in order to increase the transparency of analysis using the endline data that was collected a year after registration. Deviations from the original registered protocol include: (1) conducting difference in means rather than difference-in-difference analysis due to the lack of baseline; and (2) using an eligibility criterion of households having children < 5 years at endline, rather than <24 months at baseline (these are roughly equivalent since the intervention took 2 years and endline survey took place 1 year after intervention completion).

### Study design

From June 2009 to August 2010, the Handwashing With Soap (HWWS) and Total Sanitation and Sanitation Marketing (TSSM) campaigns were rolled out in 10 districts of Tanzania following a factorial experimental design. The sample included 181 rural wards that were randomly assigned to 4 groups receiving: the HWWS intervention alone, the TSSM alone, both the handwashing and sanitation (combination), or neither intervention (control). A baseline was planned for early 2009 but was aborted due to logistical field challenges. Random assignment of the interventions was closely adhered to during program implementation, and a cross-sectional endline survey was collected from May to December 2012, approximately one year after the conclusion of the program.

The 10 intervention districts were selected by the Ministry of Water (MoW) and Ministry of Health and Social Welfare (MoHSW) to provide geographic diversity at the national level. These districts were purposively targeted because of operational feasibility for program implementation, taking into account the existence of ongoing MoW and MoHSW projects. Of the 242 rural wards in these 10 districts, 10 wards were pre-selected as pilot areas for the program and excluded from the evaluation. Among the remaining 232 wards the program selected the 190 largest wards by population size in order to maximize the population under treatment. These were randomized into four groups of 47 or 48 wards. After randomization, the district of Masasi experienced a re-districting process through which 9 wards were reassigned to a neighboring district which was not part of the program and were dropped from the sample. The reassignment was balanced across treatment arms and included 3 wards from the TSSM-only group and 2 wards from each of the other study arms resulting in a final experimental sample of 181 wards.

**Fig 1:**
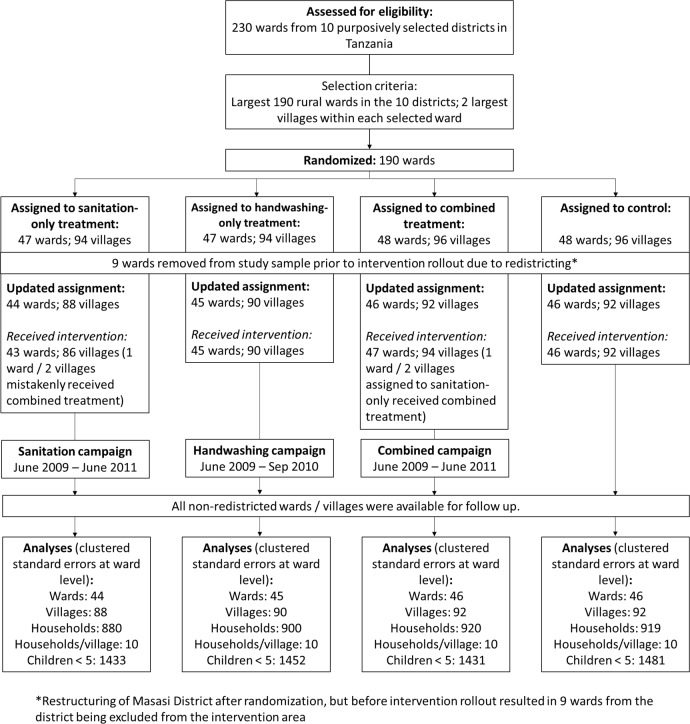
CONSORT flowchart—Enrollment, intervention coverage, and analysis of participants.

The within-ward sampling procedure followed program operational guidelines targeting the two largest villages in each treatment ward, based on population size, in each of the 181 evaluation wards. In our sample, wards had between 3 and 58 villages, with an average of 17. For the 362 villages in the sample, the full list of census enumeration areas (EAs) was obtained from the Tanzanian National Bureau of Statistics. For each village, the sample included one EA, selected with probability proportional to size. Villages in the sample wards had between 1 and 22 EAs with an average of 1.9. A household listing exercise was then conducted in each EA to collect basic information to determine household eligibility for the survey. Survey eligibility criteria were that households: (i) had been living in the village since the beginning of 2009 or earlier; and (ii) had at least one child under the age of five. Ten eligible households were then sampled from each EA at random for the survey. Fig 1 provides a graphical depiction of the sample selection process.

### Intervention description

The HWWS intervention targeted rural mothers with children under 5 with a package of intensive social marketing interventions. The campaign was built around an overarching communication concept embodied by the slogan *Mikono Yenye Fahari* (“Hands to be proud of”), which was designed to tap into mothers’ aspiration for recognition for the work they do for families. The objective was to promote behavioral change by increasing awareness of the importance of handwashing and providing technical assistance to build handwashing stations with local materials called “tippy taps”. Community “front-line activists”, or FLAs were trained to spread the message of the campaign through face-to-face interactions and help households build tippy taps. Traveling road shows and mass media radio campaigns reinforced the messages through entertaining performances. FLA’s were selected as volunteers from each community, provided with a 3-day training and required to do household visits to promote the messages of the campaign. They also distributed promotional materials and conducted handwashing promotion events with women on market days, during pre-natal clinic visits, and at village meetings.

The TSSM intervention included a package of marketing efforts similar to the HWWS campaign. The campaign focused on marketing hygienic latrines as something to aspire to, with the head of the household being the primary target. All activities within the sanitation marketing campaign fell under the common slogan *Choo bora chawesekana* (“a good latrine is possible”). These activities were coupled with a community-led total sanitation triggering event geared towards increasing demand for improved sanitation facilities and promoting open defecation free (ODF) communities. The project trained local Community-Led Total Sanitation (CLTS) trainers at the district level who, in turn, trained CLTS facilitators in each ward. After the triggering event, a CLTS committee would be formed which consisted of 5 members who were responsible for starting a village latrine register and monitoring progress, while continually motivating households to move up the sanitation ladder. This was complemented with supply side interventions to train local masons in latrine construction and marketing. The training lasted a week and taught masons how to build sungura cement sanitation platforms to be sold for approximately USD 5 per slab. In both cases, sanitation marketing messages concentrated on positive aspirational messages rather than shame tactics. No subsidies were used.

WSP worked in collaboration with Tanzania’s Ministries of Health and Social Welfare to develop the TSSM and HWWS campaign materials and trained district government counterparts who in turn conducted training of local FLAs, CLTS facilitators and masons and coordinated the distribution of marketing materials. Direct Consumer Contact (DCC) events were implemented by private companies.

Program outputs, as measured through project monitoring activities, are summarized in S3 Table to reflect the scale of the program. A more detailed description of the intervention can be found in the original working paper [17].

### Data collection

A census of households was conducted in each EA selected for the study sample. The within-EA census collected data through a listing survey on 51,091 households in the 362 selected EAs. 32.6% of listed households were eligible for the survey, primarily based on the presence of a child under age five in the household. Field supervisors then ran an in-field, automated randomization procedure to select the 10 households at random from each EA to participate in the household survey together with a replacement sample of 5 households to be used in the case of potential non-response. If a household refused to participate or was not available after two visits, the first household in the replacement sample was selected. A total of 105 such replacements were made resulting in 3619 completed interviews from 3724 attempted (97.2% response rate).

Two of the ten sampled households in each village were randomly selected for structured observations, resulting in a sub-sample of 724 households. Following the approach outlined in [18], structured observations consisted of a three hour visit to the household in the early morning (determined by the time the primary caregiver would wake up) where the enumerator would observe caregiver handwashing behavior. This was conducted before any household interviews were held in the village, with the intention of reducing any potential Hawthorne effects that could result from respondents being aware that the intention of the visit was to record handwashing behavior. Enumerators recorded the various handwashing “critical junctures” experienced by the caregiver and child, which include (i) before preparing, serving or eating food and (ii) post-toileting. They then recorded whether critical junctures were accompanied by handwashing with soap and water.

The household questionnaire included modules on demographics, productive activities and assets, water and sanitation status (observed and self-reported), education, hygiene practices and knowledge, social capital, self-reported exposure to the program interventions and child health. Health results were collected for children between the ages of 6 months and 5 years. This included caregiver-reported information from the household surveys on diarrhea and biometric markers (hemoglobin levels to test for anemia and anthropometric measurements to calculate height-for-age, weight-for-age and head circumference). The listing survey collected caregiver-reported diarrhea on the substantially larger sample size of 34,045 children under 5, increasing statistical power for the primary outcome.

### Outcome definitions

The primary and secondary indicators of interest collected through household surveys, biomarkers and structured observations are defined as follows:

#### Access to an improved latrine

The variable construction follows the Joint Monitoring Program (JMP) improved sanitation definition based on observed latrine type and private ownership. For households with latrines, we asked when the latrine was constructed to determine whether this was done during the intervention period. While a small number of households may have had flush/pour facilities, including septic tanks, we refer to “latrine” construction since 97.5% of households with a toilet facility referred to some form of pit latrine.

#### Open defecation

This refers to households that report using “no facilities” as the usual defecation practice (as per JMP guidelines.)

#### Safe disposal of child feces

This follows JMP guidelines and includes households that dispose of child feces in a latrine.

#### Open defecation-free villages

This is a self-reported measure from the community leader in the village collected during the village-level survey asking whether their village had been declared free of open defection.

#### Caregiver handwashing practices

These were collected through structured observations. We measure whether the caregiver used water and soap to wash her own or her child’s hands in conjunction with the following exposure events:

- **After fecal contact:** (i) after defecating; (ii) after toileting; (iii) after cleaning child post-toileting
- **Before handling food:** (i) before cutting or preparing food; (ii) before eating; (iii) before serving food; (iv) before breastfeeding.

#### Handwashing knowledge index

A score from 0 to 1, which represents the proportion of 5 unprompted handwashing junctures of which the caregiver is aware–after going to the latrine; after washing baby’s bottom; before preparing food; before eating; before feeding/breastfeeding.

#### Child cleanliness

We combine three binary indicators from enumerator observations (whether the child has dirty hands, fingernails or face) and average these to generate an index from 0 (dirty) to 1 (clean).

#### Caregiver hand cleanliness

Enumerators rate cleanliness on a scale of 1 (visible dirt), 2 (unclean appearance), or 3 (clean) for nails, palms and fingerpads of caregivers separately. The scores are summed to get a value between 3 and 9.

#### Diarrhea

From the household survey we use a caregiver-reported symptom-based measure defining 7 day diarrhea prevalence as having 3 or more loose/watery stools in a 24 hour period or having a stool with blood or mucus [19]. In the listing survey we ask caregivers of 34,045 children (under 5 years) a question on whether children have had diarrhea in the past 14 days in order to ensure consistency with the 2010 Demographic and Health Survey.

#### Anemia

Blood hemoglobin levels were collected in real time using a HemoCue^TM^ Hb 201+ photometer to test for anemia (iron deficiency). These biomarkers were collected for all children between 6 months and 5 years old in the household. In line with WHO guidelines, a child is considered anemic if their hemoglobin concentration is below 110 g/L [20].

#### Malnutrition

Child height, weight and head circumference were collected by specially trained enumerators with a nursing background, who followed WHO anthropometric data collection protocols to assess malnutrition levels. Results are transformed into height-for-age, weight-for-age and head circumference-for-age standardized z-scores based on WHO international growth standards [21].

#### Summary indices

Given the multitude of outcomes analyzed, we generate aggregate indices of all variables analyzed in the paper to provide a high-level outline of results. This covers the 3 main domains: sanitation intermediate outcomes, hygiene intermediate outcomes, and health impacts. For sanitation this includes access to an improved latrine, open defecation and safe disposal of child feces indicators described above. For hygiene this includes the handwashing knowledge, child cleanliness, and caregiver hand cleanliness indices. For health we include diarrhea (7 day prevalence), anemia and the anthropometric measures outlined previously. One exception is that, since aggregation is only possible for variables with the same unit of analysis, we exclude the village-level indicator on whether the village has been declared open defecation free in the sanitation index. Similarly we exclude observed handwashing behavior in the hygiene index and 14-day diarrhea collected during listing in the health index.

### Sample size

As part of the study design, power calculations were conducted for the diarrhea outcome assuming a prevalence of 0.086 and intra-cluster correlation of 0.105 based on evidence from similar study [22]. With power of 0.8 and significance of 0.05, a minimum detectable effect size of 5.4 percentage points in diarrhea prevalence rates between treatment pairs was detectable with 45 wards per treatment arm and 20 observations per ward. Ex post power calculations can be done using the control group data, yielding an intra-cluster correlation of 0.028 and mean diarrhea prevalence of 8.6%. With 45 clusters of 31 children on average in each ward, the study is powered at 80% to detect a 3.6 percentage point reduction in diarrhea between pairs.

### Randomization and masking

The ward-level randomization was stratified by district and population size using Stata. Sanitation and handwashing implementation partners were provided with the list of selected wards in mid-2008 for their respective interventions, while the list of control wards was retained by the study investigators. It was not possible to blind participants, although they were never told explicitly about the link between the survey and interventions, and any questions on program exposure were included only at the end of the survey. To mitigate enumerator bias, survey firms were never provided information on treatment status of participating wards.

### Statistical methods

We utilize the intention-to-treat (ITT) estimator as the difference between average outcomes across treatment and control groups. For both binary and continuous outcomes we calculate the least squares estimate using a linear probability model. The basic specification is:

$$Y_{ij}=\alpha +\sum_{k=1}^{3}\beta_{k}T_{jk}+\sum_{l=1}^{48}S_{jl}+\epsilon_{ij}$$ (1)

Where, *Y*_*ij*_ is the outcome of interest for household or individual *i* in ward *j*, *T*_*jk*_ is a dummy variable equal to 1 in wards assigned to receive treatment *k* where *k* = {1,2,3} for HWWS, TSSM and the combined intervention respectively. *β*_*k*_ is the estimate of the average effect of treatment *k*. The random assignment of wards was stratified by ward size within districts resulting in 48 strata or blocks. *S*_***jl***_ is a dummy variable equal to one if ward *j* is included in block *l*, representing a block fixed effects for the 48 stratified ward blocks included to improve precision [23]. For child-level outcomes we include a dummy variable for gender (male = 1) and 59 additional dummies to represent the age (in months) of the child under 5. Standard errors are clustered at the ward level. We then check whether the impacts of the three treatment arms are statistically different from each other by presenting F-statistics to test whether we can reject the null hypotheses: *β*_*1*_ = *β*_*2*_; *β*_*1*_ = *β*_*3*_ and *β*_*2*_ = *β*_*3*_. Since we run three independent tests for each outcome of interest, we also calculate and present the F-statistic for joint significance of the three treatment dummies to account for multiple hypothesis testing. The p-values associated with these tests are reported in all of the regression tables.

To provide an overview of the results, we follow [24] by generating indices for each main area of analysis: sanitation intermediate outcomes, hygiene intermediate outcomes, and health impacts. We do this by first standardizing individual outcomes (which are presented individually later in the paper) and adjusting their signs to ensure that an increase is equivalent to a positive result (e.g. switching the sign for a reduction in open defecation). Standardized z-scores are generated by subtracting the mean and dividing by the standard deviation of the control group to produce scores with a mean of 0 and a standard deviation of 1. We then aggregate all outcomes within the same index. This approach has been shown to increase statistical power for outcomes in the same domain [24].To account for the fact that the intracluster correlation varies across measures, we weight each variable by the inverse of its respective design effect.

Finally, we run two robustness checks for all the results. The first includes control variables to reduce residual variance and account for any baseline imbalance. We use the variables found to be unbalanced across intervention groups. This includes: (i) whether the household had a pit latrine or ventilated improved pit latrine (VIP) at baseline; (ii) whether household members listen to the radio; (iii) household asset ownership; and (iv) whether the household has access to piped water. Our second robustness check estimates the local average treatment effect for receiving the program, by instrumenting random assignment on actual implementation based on program monitoring data collected by dedicated project staff that collected implementation data throughout the intervention period. These additional specifications are presented in S4 Table. All analysis has been done using Stata 13.

## Results

### Study population

Since the study lacks a baseline, we use descriptive statistics from the control group to describe study population. These statistics are presented in Table 1. Households had 4.9 members on average and 75% of household heads had attended some schooling. Migration is low in our sampled areas. The majority of household heads were born in the village (68%) and those that were born elsewhere had lived in their current village for the last 17 years. Just under half of all households had access to an improved latrine and 23.1% of household members practiced regular open defecation. The predominant source of drinking water was from a well or borehole (37%) or surface water (42.2%). While 48.4% of households had some sort of handwashing device, only 12.7% of observed activities that involved household members interacting with fecal matter were followed up by washing hands with soap. Less than 1 in 50 cases where people handled food were preceded by handwashing with soap. On average, 8.6% of children under 5 experienced a diarrheal episode in the past 7 days, the height-for-age z-score was -1.1 and weight-for-age z-score was -1.03.

**Table 1:** Descriptive statistics.

Variable	n	Mean
*General household characteristics*		
Household size at baseline	919	4.89
Household head ever attended school	919	75.6%
Self-employed agricultural activities provides main source of income	919	74%
Household uses electricity as main energy source for lighting	919	2.8%
Main fuel used for cooking is firewood	919	91.0%
Household head was born in this village	919	67.8%
Number of years household head has been living in this village (if not born there)	295	17.56
*Latrine coverage and use*		
Household members use an improved latrine	919	49.7%
Household members usually defecate in the open	919	23.1%
*Access to Water*		
Main source of drinking water comes from a well or borehole	919	37.0%
Main source of drinking water comes from surface water	919	42.2%
*Handwashing Behavior*		
Household has a handwashing device	919	48.4%
Household member observed washing hands with soap after fecal contact	236	12.7%
Household member observed washing hands with soap before handling food	605	1.3%
*Health*		
Child under 5 experienced diarrhea in past 7 days	1472	8.6%
Height-for-age z-score	1323	-1.10
Weight-for-age z-score	1302	-1.03

### Randomization balance

A full table of randomization balance tests is available in S1 Table. This consists of a combination of time-invariant indicators and retrospective responses asked in the endline dating to February 2009 –before the intervention had started. Of the 87 variables we are able to present for each of the three groups (resulting in 261 comparisons to the control), we find a statistically significant difference in 12 tests for balance at the 5% significance level. The expected number of “by-chance” imbalances from a random draw of 261 is 13, which suggests that this is well within the expected range. However, some concerns persist. Firstly, half (6) of the imbalances are found in the HWWS group, which is more likely to have a cement floor and piped water connection. The sanitation only households are more likely to have a pit latrine with slab or VIP in 2009, and the combination ward households are more likely to listen to the radio and have slightly older household members than the control group.

### Compliance

Of the 181 wards selected for the sample, 45 were assigned to handwashing, 44 to sanitation, 46 to the combined intervention and the remainder to control. According to administrative records, the implementing agency accidentally conducted handwashing promotion in one of the sanitation wards, resulting in actual delivery of TSSM only to 43 wards and combined TSSM and HWWS to 47 wards. There were no reported deviations from the planned implementation of mason or FLA training, and no information was available to assess the actual delivery of village-level media (wall drawings, posters, etc.), although, based on information from the field managers, we expect that a majority of the print material dissemination was implemented as scheduled. Further details on the intervention outputs and compliance can be found in S2 and S3 Tables.

Administrative records of program implementation determined that some wards received only partial treatment. Two of the 45 HWWS wards were not exposed to the DCC event, 7 of the 43 TSSM wards did not have a CLTS triggering, and 5 of these 7 wards did not receive the DCC roadshow event. Of the 47 wards that were assigned to both TSSM and HWWS, 4 wards did not receive a CLTS triggering or DCC exposure. While our main specification yields an ITT estimate, we show in the robustness checks found in S4 Table that the results do not change signs, significance, or conclusions when using the randomized assignment as an instrumental variable for actual program implementation.

### Overview of results

Given the relatively large number of outcome variables under consideration, we use aggregate indices to present a high-level overview of results to guide the interpretation and discussion of more detailed results later in this section.

The results summarized in Table 2 present a clear and consistent story. We find significant positive impacts of the TSSM intervention on the sanitation index (sanitation intermediate outcomes), and of the HWWS intervention on the hygiene index (hygiene intermediate outcomes). Significant impacts on both the sanitation and hygiene indices are found for the combined intervention wards, with no evidence of an interaction effect. The health impacts index is indistinguishable from zero in all groups. The fact that the TSSM wards show no impact in the hygiene index and HWWS wards show no impact in the sanitation index provides for a strong falsification test that strengthens the consistency and veracity of the results.

**Table 2:** Summary indices for sanitation, hygiene and health impacts.

VARIABLES	Sanitation index	Hygiene index	Health index
	(1)	(2)	(3)
HWWS (β1)	0.029	0.096***	0.019
	(0.036)	(0.028)	(0.013)
TSSM (β2)	0.131***	0.008	0.003
	(0.035)	(0.027)	(0.012)
HWWS + TSSM (β3)	0.093***	0.108***	0.017
	(0.033)	(0.027)	(0.013)
*F-test (p-values)*			
β1 = β2	0.004	0.002	0.153
β1 = β3	0.057	0.684	0.860
β2 = β3	0.262	0.001	0.251
β1 = β2 = β3 = 0	0.001	0.000	0.303
*Observations*	3,466	3,371	5,197
*R-squared*	0.246	0.090	0.083
*Control Mean*	-0.001	0.010	-0.002

Notes: Robust standard errors in parentheses clustered at the ward level.

*** p<0.01.

*Control mean* in the final row represents the mean level of the control group for the relevant outcome. Coefficients estimated using a linear probability model including block fixed effects. Indices represent the average normalized z-score across variables used in each set (sanitation, hygiene, health), weighted by the inverse of the design effect resulting from the unique intracluster correlation for each variable. Variables that are collected at a level different to the household are not included in the sanitation or hygiene index. The same holds for health variables not collected at the child level.

### Sanitation infrastructure and behavior

Table 3 presents program impacts on latrine construction, use of improved sanitation facilities and prevalence of open defecation. The TSSM intervention produces an 8.2 percentage point increase in the probability of building a new latrine in the TSSM-only wards (95% CI 1.5–14.9; control mean = 57.1%), and a 7.7 percentage point increase in the combined wards (95% CI 1.6–13.8); these two effects are not significantly different from one another. The effect in handwashing only wards is not significantly different from the control group.

**Table 3:** Sanitation improvements.

VARIABLES	Household has a latrine that was constructed within the last 3 years	Household members use an improved latrine	HH members usually defecate in fields/bushes/rivers	Child feces are safely removed	Village has been declared open defecation free
	(1)	(2)	(3)	(4)	(5)
HWWS (β1)	-0.015	0.046	-0.032	0.043	0.046
	(0.035)	(0.034)	(0.040)	(0.036)	(0.047)
TSSM (β2)	0.082**	0.151***	-0.120***	0.117***	0.127***
	(0.034)	(0.035)	(0.039)	(0.034)	(0.049)
HWWS + TSSM (β3)	0.077**	0.106***	-0.074*	0.084**	0.087*
	(0.031)	(0.031)	(0.038)	(0.033)	(0.049)
*F-test (p-values)*					
β1 = β2	0.006	0.003	0.020	0.036	0.169
β1 = β3	0.006	0.058	0.264	0.248	0.485
β2 = β3	0.892	0.161	0.202	0.332	0.506
β1 = β2 = β3 = 0	0.003	0.000	0.012	0.004	0.043
*Observations*	3,469	3,619	3,616	3,619	362
*R-squared*	0.121	0.152	0.229	0.186	0.021
*Control Mean*	0.571	0.497	0.231	0.716	0.054

Notes: Robust standard errors in parentheses clustered at the ward level.

*** p<0.01

** p<0.05

* p<0.1.

*Control mean* in the final row represents the mean level of the control group for the relevant outcome. Coefficients estimated using a linear probability model including block fixed effects.

The increase in latrine construction in TSSM and combination wards translates not only into more latrines but also better quality ones. The probability of using improved sanitation as household members’ main method of defecation increases in both TSSM and combined treatment wards by 15.1 (95% CI 8.2–22; control mean = 49.7%) and 10.6 percentage points (95% CI 4.5–16.7) respectively.

Furthermore, the evidence supports the assertion that the TSSM intervention succeeded not only in increasing latrine construction, but also in changing sanitation behaviors by measure of open defecation prevalence and child fecal matter disposal methods. We observe large and significant reductions in open defecation in both the TSSM and combination groups. While 23.1% of households report open defecation as the primary form of feces disposal in the control group, this is 12 percentage points lower in TSSM only wards (95% CI -19.6 to -4.3) and 7.4 percentage points lower in combination wards (95% CI -14.8 to 0). Similarly, we observe statistically significant increases in the probability of correct child feces disposal, as per the JMP definition, in both TSSM and combination wards, but not within the HWWS group. Consistent with the findings on latrine construction and household open defecation, we find a significant increase of 12.7 (95% CI 3.1–22.3) and 8.7 percentage points (95% CI -0.9 to 8.7) in TSSM and combination villages respectively from 5.4% of control villages claiming to be ODF. There is no significant increase in the proportion of ODF HWWS villages. A related study [25] combines these results with detailed costing data for the interventions to estimate the cost-per-household gaining access to improved latrines as a result of the sanitation intervention which is approximately USD 194 at 2012 prices.

### Handwashing knowledge, practices, and cleanliness

We now turn to the impacts on handwashing related outcomes presented in Table 4. Caregivers in the handwashing and combined groups show small but significant improvements in knowledge.

**Table 4:** Handwashing and hygiene.

VARIABLES	Knows when to wash hands (index)	Household has a handwashing device	Household has a fixed handwashing device	Observed HWWS after fecal contact	Observed HWWS before handling food	Caregiver hand cleanliness index	Child cleanliness index
	(1)	(2)	(3)	(4)	(5)	(6)	(7)
HWWS (β1)	0.045**	-0.015	0.017**	-0.028	0.016*	0.403**	0.077**
	(0.018)	(0.039)	(0.007)	(0.030)	(0.010)	(0.187)	(0.030)
TSSM (β2)	0.007	0.025	-0.001	-0.056*	0.009	0.037	0.025
	(0.017)	(0.040)	(0.006)	(0.031)	(0.008)	(0.181)	(0.028)
HWWS + TSSM (β3)	0.040**	-0.004	0.028***	0.003	0.016*	0.455**	0.069**
	(0.017)	(0.039)	(0.008)	(0.029)	(0.008)	(0.179)	(0.030)
							
*F-test (p-values)*							
β1 = β2	0.031	0.285	0.028	0.297	0.490	0.020	0.061
β1 = β3	0.776	0.776	0.259	0.224	0.976	0.740	0.790
β2 = β3	0.049	0.439	0.001	0.031	0.456	0.008	0.118
β1 = β2 = β3 = 0	0.020	0.752	0.001	0.132	0.141	0.008	0.027
*Observations*	3,614	3,419	3,419	961	2,238	3,606	5,585
*R-squared*	0.072	0.352	0.040	0.074	0.034	0.091	0.210
*Control Mean*	0.302	0.484	0.012	0.127	0.013	6.765	0.566

Notes: Robust standard errors in parentheses clustered at the ward level.

*** p<0.01

** p<0.05

* p<0.1.

*Control mean* in the final row represents the mean level of the control group for the relevant outcome. Coefficients estimated using a linear probability model including block fixed effects.

The intervention does not have an impact on the probability of having any form of handwashing station, including mobile stations (48% of households do), but does have a relatively large impact on the probability of having a fixed handwashing station. While only 1.2% of control households have a fixed handwashing station, this proportion increases by 1.7 (95% CI 0.3–3) and 2.8 (95% CI 1.2–4.4) percentage points respectively in HWWS and combination groups. Most of this change is driven by the presence of “tippy taps”, which we classify as fixed handwashing stations.

Overall, we find no improvements in handwashing with soap after fecal contact, but small (borderline significant) increases in observed handwashing practice before preparation of foods. There is a 1.6 percentage point (95% CI -0.4 to 3.6) higher likelihood of observing handwashing when handling food or feeding among members of the HWWS and combination groups, compared to a mere 1.3% of handwashing observed at this junction in the control group. When accounting for multiple hypothesis testing through the joint F-test, we fail to reject the null hypothesis that observed handwashing practices are unchanged across all intervention groups.

Overall, children appear to be cleaner in the HWWS and combination wards than in the control or TSSM wards. The cleanliness index is 0.566 for children in the control group, and shows improvements of 0.077 (95% CI 0.018–0.136) and 0.069 (95% CI 0.011–0.129) points respectively in the handwashing and combination wards.

The results are consistent for each individual indicator comprised in the index. The caregiver hand cleanliness index also shows improvement in handwashing and combination wards. We observe an improvement of about 0.4 (95% CI 0.03–0.77) and 0.45 (95% CI 0.1–0.81) points in the handwashing and combination wards respectively, above a mean score of 6.7 in the control group. No changes in cleanliness are observed in TSSM wards.

### Health outcomes

The TSSM and HWWS interventions are aimed at provoking handwashing and sanitation related behavior change with the ultimate goal of improving the population’s health, particularly health outcomes amongst children under 5. These results are presented in Table 5.

**Table 5:** Health.

VARIABLES	Diarrhea in past 7 days	Diarrhea in past 14 days (Listing Data)	Hemoglobin level (g/L)	Anthropometric z-scores		
	Weight-for-age	Height-for-age	Head circumference
	(1)	(2)	(3)	(4)	(5)	(6)
HWWS (β1)	-0.004	-0.013	0.089	0.015	0.030	0.229
	(0.012)	(0.011)	(0.754)	(0.043)	(0.057)	(0.141)
TSSM (β2)	-0.001	-0.010	-0.772	-0.044	-0.006	0.092
	(0.012)	(0.012)	(0.713)	(0.035)	(0.059)	(0.129)
HWWS + TSSM (β3)	-0.011	-0.021*	-1.652**	-0.075**	-0.008	0.227
	(0.013)	(0.013)	(0.772)	(0.038)	(0.057)	(0.154)
*F-test (p-values)*							
β1 = β2	0.744	0.813	0.208	0.137	0.515	0.263
β1 = β3	0.594	0.428	0.023	0.039	0.476	0.991
β2 = β3	0.399	0.360	0.214	0.396	0.961	0.351
β1 = β2 = β3 = 0	0.807	0.416	0.085		0.096	0.884	0.307
*Observations*	5,768	34,045	5,203	5,203	5,208	5,208
*R-squared*	0.053	0.051	0.194	0.062	0.084	0.223
*Control Mean*	0.086	0.168	111.441		-1.033	-1.946	-0.511

Notes: Robust standard errors in parentheses clustered at the ward level.

** p<0.05

* p<0.1.

*Control mean* in the final row represents the mean level of the control group for the relevant outcome. Coefficients estimated using a linear probability model including block fixed effects, child gender and age (month) dummies. Column (1) reports on the symptom-based diarrhea measure captured in the household survey. Column (2) reports on direct diarrhea reports captured in the listing survey.

The 14-day diarrheal recall collected during listing (n = 34,045) registers insignificant effects in the TSSM and HWWS wards and a borderline significant decline in diarrhea of 2.1 percentage points (95% CI -0.4 to 4.6) in the combined treatment group, compared to a control group mean of 16.8%. When we analyze the caregiver-reported outcomes on diarrhea symptoms in the past 7 days from the household survey we observe no significant differences between treatment and control groups.

We observe no differences in the level of measured Hb for children in the TSSM and HWWS-only groups. However, in the combined treatment group, children show a small but significant decline of 1.65 g/L (95% CI 0.14–3.17) in hemoglobin (a 1.5% relative decline compared to the average of 111.4 g/L in the control group). A second indicator that does not change in the singular treatment arms is weight-for-age (z-score). However, the average weight-for-age z-score in the combined intervention group declines by 0.075 standard deviations (95% CI -0.15 to 0) off of an average weight-for-age z-score of -1.03, which is statistically significant.

Finally, our indicators for long run child health are the height-for-age z-score and head circumference-for-age z-score. On average we find no effects of the program on the height of children in any of the treatment groups relative to control and estimated coefficients are close to zero. For head circumference, estimated coefficients are positive but not significant with relatively large standard errors driven by a higher intracluster correlation than height and weight measures.

## Discussion

Four systematic reviews over the past decade have shown that handwashing with soap consistently reduces diarrhea by between 39% and 47% [14], [26], [27], [28]. This evidence, however, comes mostly from small-scale efficacy trials exploring proof of concept, or matched studies with high levels of handwashing compliance. While promising, it is not clear how scalable these interventions are, and thus how much national policy can and should draw from this evidence when designing at-scale programs where handwashing compliance is more limited. Apart from recent work in India [29], most of the available research suggests that, when taken to scale, sustained behavior change for meaningful health improvements remains elusive—e.g. [4] and [5].

Evidence on the effectiveness of at-scale rural sanitation interventions is mounting, and suffers from a similar challenge to handwashing programs–limited take up leading to the majority of completed randomized controlled trials (RCTs) finding no consistent health improvements [6], [7], [8]. One explanation for the limited health improvements observed in the literature focuses on the *intensive* margin of fecal-oral transmission–at-scale programs often fail to achieve the level of coverage and use necessary to effectively reduce fecal-oral pathogen transmission. Recently published work in Mali provides support to this view [11]. The intervention increases latrine coverage from 66% to 90% and finds significant improvements in height-for-age. However, a study of the Total Sanitation Campaign in India also identified a successful increase in latrine coverage from 9% to 60% versus a negligible change in the control group but found no health impacts, suggesting that even with substantial improvements in latrine coverage, health improvements are not guaranteed [7]. In this case, less than two thirds of facilities were actually used, highlighting the potential of improved behavior change accompanying increased coverage. An alternative, but largely untested hypothesis for observing limited health impacts in individual handwashing and sanitation campaigns focuses on the *extensive* margin of fecal-oral transmission–blocking off multiple pathways at once. If improvements in intermediate outcomes for at-scale sanitation and handwashing campaigns are difficult to achieve, and even when achieved, are not guaranteed to produce meaningful health improvements on their own, a natural extension to the current evidence is to understand the potential complementarities of these interventions as a more holistic approach to cutting off disease transmission.

Our study finds intermediate results in line with the current evidence for at-scale interventions in hygiene and sanitation, suggesting that sanitation improvements are easier to generate than handwashing behavior change [30]. The TSSM intervention that we study in Tanzania increased the coverage of improved sanitation by 15.1 percentage points while reducing open defecation by 12 percentage points which is slightly larger than the mean effects on sanitation coverage and use found in a recent review [12]. The handwashing intervention results are more modest, increasing caregiver knowledge of appropriate handwashing times and improving child and caregiver cleanliness, but generating no meaningful changes in actual handwashing behavior. Promoting behavior change that requires once-off actions are generally more successful than ones that require change in daily behaviors [31]. It is therefore unsurprising that using promotion to increase latrine coverage was found to be more effective than trying to change handwashing practices. While some novel behavioral “nudges” have been shown to successfully increase handwashing in the short term, it is still unclear how effective these treatments may be in the longer run [32]. In the HWWS program, tippy taps were meant to be a key behavioral driver for improved handwashing by making washing hands with soap easier and more salient. However, program reports suggest that, while many people constructed tippy taps early on, they soon broke and only 22 were observed across the entire sample at endline.

Even when combining interventions we find no health improvements for children under 5. At an estimated cost of 194 USD per household gaining access to improved sanitation, and limited observed changes in hygiene behavior, the results suggest that the types of changes possible by relying purely on a well-financed behavior change approach in this context do not seem adequate for generating meaningful health improvements, even when combining sanitation and handwashing promotion.

## Limitations

The study lacks a baseline of pre-intervention characteristics. A baseline was commissioned in 2009 before the interventions began, but half way through the data collection it became clear that the survey firm was not able to deliver reliable data. Rather than invest in a second baseline survey that would have overlapped with the intervention start date, the research team chose to focus resources on the endline given that the randomized intervention allocation went according to plan and the sample was large enough to be balanced in expectation. While we attempt to reconstruct baselines for key variables, these suffer from problems of recall. Since imbalance is reported in a few key sanitation variables, a potential concern is that results in the TSSM group may be driven by some true baseline imbalance. Controlling for baseline latrine type, we find positive TSSM results reduce slightly and converge to be more closely aligned with combination ward results, with signs and significance tests remaining the same with and without controls. These results are presented in S4 Table.

Differential migration and attrition could also be a concern. However, data from the complete census listings of selected enumerator areas provides evidence of limited migration and attrition. Less than 5% of households moved into the community within the three-year intervention period, and this does not differ across treatment groups. We mitigate any confounding that migration may cause by restricting the sample to households residing in the area since 2009.

Sanitation and hygiene studies in the past have suffered from potential self-reporting bias [33]. This may be the case for a number of our outcomes since we rely primarily on caregiver-reported measures such as self-reported diarrhea; however, we complement this with biomarkers for the main health outcomes of interest to mitigate this problem.

Although the study covered a large part of the country, the study areas were purposively selected, limiting external validity. However, comparing our control group in the 10 study districts to representative surveys of rural Tanzania, including the 2010 Demographic and Health Survey and National Panel Survey, reveals that the two areas are more similar than not along basic observable characteristics (the comparison can be found in S5 Table) which strengthens the potential relevance of these findings for other regions of the country. Given the important implementation challenges of promotion campaigns, the results may be more relevant to other large-scale government-led interventions than smaller-scale, more tightly-controlled activities.

## Conclusion

Moving beyond efficacy trials, this study explores what can be achieved *at scale* through independent and combined sanitation and handwashing campaigns and builds on evidence from the global WSP program looking at the impacts of government-led, at-scale interventions in Vietnam [4] and Peru [5] (HWWS), and Indonesia [6] and India [8] (TSSM).

The results from this study highlight the importance of focusing on intermediate outcomes of take up and behavior change as a critical first step before realizing the changes in health that WASH interventions aim to deliver. Finding the balance between intensity, the right incentives, holistic coordination and scale becomes an important policy question. The biological reasoning behind promoting WASH interventions is theoretically sound, but identifying ways to close the gap between objectives, intervention design and delivery, particularly when working at scale, should be a priority for researchers, policymakers and implementers alike.

## Supporting information

S1 Table Balance statistics. (XLSX)

S2 Table Program compliance. (XLSX)

S3 Table Program outputs. (XLSX)

S4 Table Robustness checks. (XLSX)

S5 Table External validity. (XLSX)

S1 Text CONSORT checklist. (DOCX)

S2 Text Study protocol. (PDF)
