# Supplementary material for: Are there synergies from combining hygiene and sanitation promotion campaigns: Evidence from a large-scale cluster-randomized trial in rural Tanzania
Source: PLoS One. 2017 Nov 1;12(11):e0186228. doi: 10.1371/journal.pone.0186228 (PMC5665426; doi:10.1371/journal.pone.0186228)
Supplement: S2 Text — (PDF) [file pone.0186228.s007.pdf]

# CONCEPT NOTE

# IMPACT EVALUATION
# OF
# INNOVATIVE APPROACHES TO
# HANDWASHING AND SANITATION IN TANZANIA


**Prepared by:**

**Sebastian Martinez**
**The World Bank**

**Alicia Salvatore**
**University of California, Berkeley and The World Bank**


**Submitted To:** **Eduardo Perez, Senior Sanitation Specialist, WSP**
**Ousseynou Diop, Senior Water and Sanitation Specialist, WSP**
**Jack Molyneaux, Senior Water and Sanitation Specialist, WSP**
**Nathaniel Paynter, Water and Sanitation Specialist, WSP**


**Submission Dates:** **December 11, 2007**
**September 25, 2007** *(preliminary version for comments)*

**Implementation Team**

| | |
|---|---|
| **Project Name** | Handwashing and Sanitation Project |
| **Country** | Tanzania |
| **Task Team Leaders** | Eduardo Perez, Ousseynou Diop, Nathaniel Paynter |
| **Global Impact Evaluation Team Members** | Jack Molyneaux, Alexandra Orsola, Paul Gertler, Sebastian Galiani, Jack Colford, Lia Fernald, Patricia Kariger, Pavani Ram |
| **Country Principal Investigators** | Sebastian Martinez and Alicia Salvatore |
| **Research Assistants** | Patrick Mwakilama |

**Tanzania Impact Evaluation Concept Design Team**

The initial impact evaluation design for the Handwashing and Sanitation Promotion project in Tanzania was developed during a two day workshop held in Dar Es Salaam, Tanzania on July 2 and 3, 2007. Contributions to the initial impact evaluation concept design were received from partners in the Ministries of Water and Health, WSP, World Bank, and other donors:

| **Name** | **Institution** | **E-mail & Phone** |
|---|---|---|
| Vivienne Abbott | Water Aid | vabbott@wateraidtanzania.org<br>0754-324246 |
| Rebecca Budima | UNICEF | rbudima@unicef.org |
| Sebastian Martinez | World Bank | smartinez@worldbank.org |
| Mary K. Mbowe | Ministry of Water | mrymbowe@yahoo.com.uk<br>0713-325776 |
| Dorisia Mulashani | Ministry of Water | saveness@yahoo.com<br>0784-299207 |
| Anyitike Mwakutalima | Ministry of Health | Anyitike76@yahoo.com.uk<br>0756-392688 |
| Kaposo Mwambuli | WSP | kmwambuli@worldbank.org |
| Nathaniel Paynter | WSP | npaynter@worldbank.org |
| Mussa Uwesa | UNICEF | muwesa@unicef.org |

**TABLE OF CONTENTS**


| | | |
|---|---|---|
| | **ACRONYMS**………………………………………………………………... | 1 |
| **I.** | **EXECUTIVE SUMMARY** ……………………………………………...……… | 2 |
| **II.** | **HANDWASHING AND SANITATION PROJECTS IN TANZANIA** …..... | 3 |
| | A. Promotion of Handwashing with Soap ……………………………….... | 3 |
| | B. Sanitation Promotion ……………………………………………...……... | 4 |
| **III.** | **PRINCIPAL HYPOTHESES AND RESEARCH QUESTIONS** ………….. | 6 |
| **IV.** | **IMPACT EVALUATION METHODOLOGY** …………………………...… | 9 |
| | A. Counterfactual Analysis …………………………………….……... | 9 |
| | B. Promotion of HW and TSSM at the local level: Randomized Design…. | 10 |
| | C. Promotion of HW and TSSM at the regional level: Quasi-experimental designs | 13 |
| **V.** | **COSTING** …………………………...……………………………………… | 15 |
| | A. Analysis of Costs ……………………………………...……….……... | 15 |
| | B. Analysis of Effectiveness ……………………….…………...….. | 17 |
| | C. Analysis of Benefits …………………………………………...….. | 17 |
| | D. Sensitivity Analysis ………………………………………………...….. | 18 |
| **VI.** | **SAMPLE DESIGN** and RANDOM ASSIGNMENT OF TREATMENT……. | 19 |
| | A. Sample Selection Stage 1 ……………………………………………… | 19 |
| | B. Sample Selection Stage 2 ……………………………………………… | 21 |
| | C. Sample Selection Stage 3 ……………………………………………… | 22 |
| | D. Sample Selection Stage 4 ……………………………………………… | 22 |
| | E. Power Calculations ……………….………………………………… | 23 |
| **VII.** | **SURVEY INSTRUMENT** and ROUTINE MONITORING DATA………… | 26 |
| **VIII.** | **IMPACT EVALUATION TIMELINE** ………….………………...………... | 26 |
| **IX.** | **APPENDICES** ………………………………...……………………… | 26 |
| | Appendix A : MAP……..……………………………………………….. | 29 |
| | Appendix B : STATA CODE……………………………………….. | 30 |
| | Appendix C : LIST OF WARDS IN T/C………………………………. | 33 |
| | Appendix D : KEY FEATURES OF IE FOR INTERVENTION……… | 35 |
| | Appendix E : EVALUATION DESIGN LOG………………...……… | 36 |

## ACRONYMS

| | |
|---|---|
| CBO | Community-Based Organization |
| DWST | District Water and Sanitation Team |
| GoT | Government of Tanzania |
| HVC | Healthy Village Campaign |
| IE | Impact Evaluation |
| MoHSW | Ministry of Health and Social Welfare |
| MoW | Ministry of Water |
| NGO | Non-Governmental Organization |
| SHWG | Sanitation and Hygiene Working Group |
| SM | Sanitation Marketing |
| TS | Total Sanitation |
| TSSM | Total Sanitation & Sanitation Marketing |
| WB | World Bank |
| WSP | Water and Sanitation Program |
| WSP-AF | Water and Sanitation Program-Africa |
| WSP-TZ | Water and Sanitation Program-Tanzania |

# I. EXECUTIVE SUMMARY

This concept note proposes an impact evaluation (IE) for the Scaling up Handwashing with Soap (HW) and Total Sanitation and Sanitation Marketing (TSSM) projects of the Government of Tanzania (GoT) with support from the Water and Sanitation Program (WSP). The concept note outlines the evaluation's objectives, methodology, data and sampling plan, work plan and timeline, and is a "working document" to guide the IE, while at the same time remaining adaptable to changes and updates as required by the projects. The concept note will incorporate input from the IE team and project partners and serve as the basis for a final IE design completed prior to fielding of the baseline survey.

The broad objective of the IE is to estimate the causal impact of the HW and TSSM interventions on the health and welfare of the rural poor in Tanzania. The IE will also, where feasible, test innovative programmatic design components to inform the GoT on operational questions that can help optimize the use of resources as the HW and TSSM approaches are taken to scale. In the context of the global Gates-funded program of HW and TSSM (including Peru, Senegal, India, Vietnam, and Indonesia), Tanzania is the only country in the wider program of evaluation to include both types of interventions in the same environment. Therefore, a key component of the IE in Tanzania is testing the effects of combined HW and TSSM interventions (interaction effects). Other elements under consideration for examination in the IE include geographic intensity, frequency of treatment, and types of HW and TSSM promotion activities.

The proposed IE uses a cluster-randomized experimental design, whereby the interventions are randomly assigned to a sub-set of intervention clusters within 10 treatment districts. The sampling process for the randomized IE design was completed in three stages. First, 10 districts[1] where chosen by the Ministry of Water (MoW) and Ministry of Health and Social Welfare (MoHSW) in agreement with the WSP (see Appendix 1). These 10 treatment districts were selected because of operational feasibility for rapid roll out of the pilot phase of the project. While the 10 selected districts present a geographically diverse set of areas, the selection was non-random[2]. Second, within the 10 treatment districts, 200 eligible wards were selected, and randomly assigned to one of four groups: (1) Handwashing intervention, (2) Sanitation intervention, (3) Handwashing and Sanitation intervention, and (4) Control (non-intervention). In a third stage, clusters of minimum-cost efficient units of intervention will be identified within the 200 evaluation wards. A random sample of 200 to 250 clusters will be selected, with 47 or 48 clusters assigned to each of the three treatment groups (47 Handwashing, 47 Sanitation, 48 Handwashing and Sanitation), and up to 100 clusters assigned to the control group.

[1] There are 129 districts in Tanzania. Source: National Bureau of Statistics Tanzania (www.nbs.go.tz).

[2] The 10 treatment districts have ongoing or planned health and water and sanitation interventions. Although specific villages forcibly included in the treatment group will be excluded from the IE sample, external validity at the district level is not guaranteed under this design.

## II. HANDWASHING AND SANITATION PROJECTS IN TANZANIA

### A. Promotion of Handwashing with Soap[3]

The handwashing with soap intervention is targeting mothers/caregivers of children under-five-years-old. Children under five represent the most susceptible age-group to the serious consequences from diarrhea and respiratory infection. They are also the least likely to benefit directly from increased sanitation coverage. Diarrheal disease and respiratory infection among children under five can be prevented by their mothers/caregivers washing their hands with soap at key times such as before feeding a child, cooking, or eating and after using the toilet or changing a child.

To increase handwashing behaviors among mothers/caregivers the handwashing intervention is based on an implementation approach which borrows from both commercial and social marketing fields. This entails designing communications and messages likely to bring about the desired behavior changes and delivering strategically so that the target audiences are "surrounded" by handwashing promotion. Some key elements of this intervention include:

- key behavioral concepts or triggers[4] for each target audience;
- a persuasive argument which analyzes why and how a given concept or trigger will lead to behavior change; and
- a communications idea, which conveys the concept through many integrated activities and communication channels.

The implementation plan will be designed using formative research and any subsequent spot research deemed necessary. A triggering workshop will be held to help the team identify messages to provoke behavioral change among mothers/caregivers. A communications/marketing firm will be contracted to guide the campaign. This firm will be expected to develop a comprehensive and integrated communication approach including a variety of communications channels, both mass media and direct consumer contact (e.g., events in markets and other areas where women gather).

The national handwashing intervention program will be phased into selected districts. The project will launch with a national event engaging the media, politicians, and other notable persons and will likely "roll out" mass media such as radio, bill boards, and clothing to bring key messages of the campaign to communities. Direct consumer contact activities, and other outreach and marketing techniques (e.g., carnivals, contests, plays, games, women's groups, and marketplace events) will be employed as well.

---

[3] Tanzania Handwashing Project Implementation Plan. June, 2007

[4] Triggers are images or messages that are designed to attract attention, raise awareness, and encourage behavior change.

## B. Sanitation Promotion[5]

Household investments in basic sanitation have become the norm since Julius Nyrere, the first president after independence, implemented a latrinisation program in the 1970s. The rapid, top-down approach employed has resulted in wide-spread latrine and also a willingness, on the part of community members, to pay for latrines. Over the last few decades, however, many of the latrines constructed in earlier sanitation efforts have fallen into disrepair. Lack of quality latrines and latrine hygiene is also a significant issue in Tanzania. The GoT/WSP Sanitation project will apply an innovative approach to address these issues and revive sanitation promotion in Tanzania. The Sanitation intervention aims to move households up or onto "the sanitation ladder" by stimulating demand for sanitation, especially quality latrines. Traditional sanitation marketing and Total Sanitation (TS) approaches will be used to these ends. The program intends to increase the current supply of latrines to meet the anticipated demand by strengthening the local private sector (e.g., building supply chains for goods, technical skills, and marketing abilities).

Sanitation Marketing (SM) can be defined as: an approach that utilizes the power of the small- and medium-scale private sector in the provision of sanitation services and uses techniques of commercial marketing to analyze the themes and messages that would generate demand for these services and lead to behavioral change. Total Sanitation (TS) focuses on improving sanitation coverage and services at the village level by highlighting the problems caused to all residents by poor sanitation and hygiene within and around the community, and by ensuring that every household builds, uses, and maintains its own low-cost toilet, or at least has access to and uses a shared toilet. This approach creates demand for sanitation by building upon a combination of peer pressure at the community level and collective action to help destitute members of the community and public facilities (schools and hospitals) obtain sanitation solutions. The generation of demand for sanitation services moves from the individual to the community level. Governments at the central and local levels support total sanitation programs by providing a "software" subsidy to cover the promotion and mobilization costs and offer village-level grants to reward achievement of the community-level open defecation–free status, which will be determined through independent certification.

For the implementation component of this project, the overall approach is to capitalize on the existing, high-levels of unsatisfactory latrines in an effort to move households up the sanitation ladder. At the moment, most of rural Tanzania is on the lowest rung - a traditional pit latrine that does not adequately isolate feces from humans. The plan is to have the households invest in retrofitting existing latrines with sanplats, which will also be incorporated into any new latrines constructed in the period. To do this, we will stimulate demand through the total sanitation approach, as well as identifying and targeting communities that are still practicing open defecation. To supply the demand, we will work with *fundis* (local artisans) on developing their skills to construct the sanplats necessary to improve the latrines. This work will be closely

[5] Tanzania Total Sanitation and Sanitation Marking Project Implementation Plan. July 2007.

integrated with that of counterparts from the Ministries of Water and Health and local government.

For the IE, the project will conduct a thorough baseline of the target area to determine the actual range of sanitation technologies, as well as current rates of diarrheal incidence. Additionally, a thorough review of existing sanitation interventions will be conducted. Emphasis will be placed on researching prior interventions that have created enabling environments, and stimulated consumer demand. Obstacles and triggers to widespread adoption of the sanitation upgrading used previously will be summarized.

The results of the assessments will inform project implementation. Although intended for national scale, implementation will begin in five districts, before being expanded to another five, and then nationwide. The 10 districts proposed for initial implementation are Mpwapwa, Kondoa, Rufiji, Iringa, Sumbawanga, Kiteto, Masasi, Musoma, Karagwe and Igunga.

## III. PRINCIPAL HYPOTHESES AND RESEARCH QUESTIONS

The IE will assess the impact of exposure to the HW and TSSM promotion on individual-level sanitation practices and on the health and welfare of children, particularly children 0-5 years old. By introducing exogenous variation in handwashing and sanitation practices (through exposure to the HW and TSSM promotion), the IE will also answer a number of important questions relating to the effect of the intended behavioral change (handwashing and improved sanitation) on health and welfare, thus providing information on the extent to which these behaviors alter intended development outcomes[6]. The IE will aim to address the following primary research questions and associated hypotheses:

1. **What is the effect of *handwashing promotion* on handwashing behavior?**
   We hypothesize that promotion of handwashing through social marketing campaigns will increase the frequency of handwashing and increase the frequency of handwashing at recommended times (e.g., after using the toilet, before preparing meals) by changing people's awareness of handwashing and provoking an increased demand for handwashing as part of daily hygiene habits.

2. **What is the effect of *handwashing promotion* on health and welfare?**
   We hypothesize that promotion of handwashing through social marketing campaigns will improve the health of the population especially children under five years old, a population that is vulnerable to intestinal and respiratory maladies transferred from dirty hands to food sources or by direct contact with the mouth. The health impact of the intervention will result from the positive behavior changes stated above (e.g. increased frequency handwashing with soap and compliance with recommended timing). Improved health in the household, in turn, improves welfare by increasing productivity and time available for productive or leisure activities, as measured by socio-economic indicators, labor market participation, and scales of happiness, stress and depression. The improved health (notably reduced diarrhea prevalence and intestinal parasites) will also promote physical, motor skill and cognitive development in young children.

3. **What is the effect of *sanitation promotion* on changes in sanitation behavior?**
   We hypothesize that promotion of sanitation through social marketing campaigns will improve the quality and coverage of improved latrines and increase recommended sanitation practices (e.g., reducing open defecation) by increasing the demand for

[6] The HW and TSSM promotion will be used as an Instrumental Variable (IV) to estimate the Local Average Treatment Effect of increased handwashing and sanitation on the health and welfare of the population. Since handwashing and sanitation are endogenously determined, the random assignment of promotion will insert exogenous variation in handwashing and sanitation behavior between treatment and comparison communities, providing an appropriate instrumental variable for estimating the effect of handwashing or sanitation on compliers.

improved facilities and meeting the demand with adequate supply through the training of local artisans.

4. **What is the effect of *sanitation promotion* on health and welfare?**

   We hypothesize that promotion of sanitation through social marketing campaigns will improve the health of the target population by facilitating improved hygiene of the toilet facilities, and thus reducing the exposure of young children to fecal matter in the environment. Improved health in the household, in turn, may improve welfare by increasing productivity and time available for productive or leisure activities, as measured by socio-economic indicators, labor market participation, and scales of happiness, stress and depression. The improved health (notably reduced diarrhea prevalence and intestinal parasites) will also promote physical, motor skill and cognitive development in young children.

5. **What are the interaction effects of providing handwashing promotion and sanitation promotion jointly?**

   We hypothesize that the joint combination of both types of intervention has at least additive effects, that is, that the presence of the two interventions produces outcomes that may be greater than the sum of the individual parts.

In addition, the IE will seek, where possible, to address a number of secondary questions, including:

6. **What are the conditions (i.e., presence of water, soap, latrines) under which the handwashing and sanitation promotion strategies are most effective in achieving desired outcomes?**

   Intervention impacts may differ depending on initial household and community characteristics. Understanding variation in program impacts according to initial characteristics (impact heterogeneity) can indicate which communities and individuals may require greater attention and assistance to produce the desired effects. This information will help improve future program design and targeting.

7. **Which promotion strategies are more cost-effective in achieving desired outcomes?**

   Impacts per unit cost may differ according to the effectiveness of the promotion strategy. Following the pilot phase of promotion design, it is proposed that two competing approaches may be tested against one another to provide guidance on the scale up options. Current proposals include: Local Mass Media, Village community events, School events. In addition, it may be possible to test the optimal combination and timing of local events with national publicity events (e.g., National media campaigns, Presidential radio addresses).

8. **What are the optimal levels of intensity of treatment (number of messages)?**

A "tipping point" in behavioral change may be provoked by an optimal exposure to promotional messages. Understanding the optimal frequency and combinations of social marketing messages will provide guidance on the optimal intervention design as the program is scaled up.

9. **What are the optimal levels of coverage (number of villages in a fixed geographical area)?**

   Informational spillovers and spread of intervention messages may provoke behavioral changes in communities within an "area of influence" adjacent to treatment clusters. It is proposed that exogenous variation in the "density of treatment" within a pre-defined geographical area will be introduced by randomizing the number of treatment clusters per Ward. Outcomes in surrounding non-intervention villages can then be compared to control villages in low-density or non-treatment Wards to analyze informational spillovers from social marketing campaigns. If informational spillovers are small, it is possible that effects would not be captured under the current sample structure due to insufficient power.

## IV. IMPACT EVALUATION METHODOLOGY

To address the proposed research question, a proper IE methodology is required to establish the causal linkages between the intervention and the outcomes of interest. This section describes the proposed methodology and its application to the Tanzanian HW & TSSM case.

### A. Counterfactual Analysis

In order to estimate the causal relationship between the HW and TSSM interventions (treatment) and the outcomes of interest, IE requires the construction of a counterfactual – that is, what would have happened to the target group in the absence of the intervention. In the case of HW and TSSM, it is possible that factors such as weather, macro-economic shocks, or other new and ongoing public health, nutrition, sanitation, and hygiene campaigns, to mention a few, could influence the same set of outcomes that are targeted by HW and TSSM (e.g., diarrhea incidence in young children, health and welfare). To account for factors external to the intervention, counterfactuals are estimated using control or comparison groups that are equivalent to the treatment group on every dimension (observed and unobserved) except for the treatment, and thus account for time varying factors that may affect the target population. Since a good counterfactual approximates what would have happened to treatments in the absence of the treatment, any differences in the average outcome measurements of treatment and control groups following the program implementation can then be attributed as the causal effect of the intervention.

Where feasible, this IE will use a randomized experiment to estimate the causal impacts of the HW and TSSM promotion campaigns on the outcomes of interest. Random assignment of treatment to a sub-set of communities can ensure that the treatment and comparison groups are equal,[7] and thus that an appropriate counterfactual can be measured. This approach is viable for intervention sub-components that are targeted at relatively disaggregated units of intervention such as the household, village or ward. For interventions that target large geographical clusters, such as district or national level media campaigns, the IE will propose alternative quasi-experimental methods.

A randomized experimental evaluation with a comparison group is valuable because it reduces the possibility that the observed before-to-after changes in the intervention group are due to factors external to the intervention. If no control group is maintained and a simple pre- to post assessment is conducted of the HW and TSSM interventions, one cannot attribute changes in outcomes to the intervention with any certainty. As discussed previously, it is possible that other changes occurring over the same time period, such as weather or economic growth and development may be the "true" causes of the observed changes, or at least contributed to the outcomes in some

[7] Technically this is only true with infinite sample sizes, which is unaffordable and unnecessary. Instead, this study seeks to minimize the risk that the means of the treatment and comparison groups differ significantly.

way. For example, if the baseline year had normal rainfall levels and the post-intervention follow up year had higher than average rainfall, we may observe a rise in the incidence of diarrhea in the population between the two years. From this simple before and after comparison, the analysis would conclude that the HW & TSSM program led to higher rates of diarrhea. However, it is likely that the increase in diarrhea may have been due to the higher than normal rainfall that increased contamination in drinking water sources, for example. By surveying a control group that does not receive the program, the evaluation can estimate the average impact of the HW & TSSM programs over time, independent of external factors such as weather, and thus avoids confusing the program impact with these other influences.

The use of a random control group also helps to prevent other problems. For example, communities that are chosen purposively as areas with a high likelihood of success for programs such as HW & TSSM because of favorable local conditions (strong leadership, existing water and sanitation infrastructure, highly educated population, etc) are likely to be different from areas that are considered less desirable for implementation. If a non-random control group is used, a comparison of treated and untreated areas would confuse the program impact with pre-existing differences, such as different hygiene habits, lower motivation, or other factors that are difficult to observe. This is known as *selection bias*. A random control group avoids these difficulties, by ensuring that the communities that receive the program are no different than those that do not.

In the following sections we outline the evaluation design for the Tanzania HW and TS program. Two major types of interventions will be implemented; these can be categorized as **local** and **regional**. Local campaigns concentrate on social marketing campaigns and mass media at the local level. While the precise geographic clustering of local interventions has yet to be defined, these are assumed to approximate an area comprised by a collection of hamlets or villages. Regional campaigns, on the other hand, are expected to stretch across larger geographic clusters, such as a collection of Wards or Districts, which could be included within an area of influence for radio media, for example[8]. The identification strategies for the HW & TSSM interventions at the local and regional levels in Tanzania are discussed in detail below.

## B. Promotion of HW and TSSM at the local level: Randomized Design

The local HW and TSSM promotion interventions will be evaluated using a randomized design. This strategy is feasible during the initial two year pilot program where funding is available for approximately 100 HW and 100 TSSM units of intervention nationally. In principal, all rural areas in Tanzania are eligible for treatment. As such, the number of eligible sites is vastly larger than the number of benefits available

[8] It is not expected that a purely experimental design will be feasible for the regional campaigns, and opportunities for evaluating regional interventions will be considered using secondary data sources or with a matched sample of primary data.

during the pilot phase[9]. Taking into consideration the operational and logistical requirements of clustering the local interventions in a set of geographically representative areas, a fair and transparent rule for allocating the benefit is to give each eligible site an equal chance of receiving the benefit. Under this design, sites (or clusters of sites) will be randomly phased into the program over time until the quota of available units of intervention is filled. This design produces treatment and control groups with roughly balanced characteristics (observed and unobserved) at baseline. Then, following the implementation of the HW and TSSM promotion in beneficiary areas, the differences in average indicators between treatment and control areas will approximate the true causal effects of the program.

Ten districts have been pre-selected for implementation of the local interventions. These districts were chosen by the evaluation team because of operational feasibility for program implementation, taking into account the existence of ongoing MoW and MoHSW projects, including the Health Village Campaign (HVC) and water and sanitation interventions. Five MoHSW HVC villages have been selected to include handwashing promotion, and five water and sanitation villages will be forcibly included under the TSSM treatment. These areas will be included in the treatment group, but will be excluded from the evaluation sample because they constitute a non-random group of villages. These 10 villages will also constitute the first areas of intervention during the program design phase. It is important to note that the ten intervention districts were originally chosen by the MoW and MoHSW to provide geographic representation at the national level, however it is unknown at the time of writing whether these constitute a representative sample of districts. Additional analysis will assess the comparability of the intervention districts with other districts at the national level.

The ten intervention districts are sub-divided into a total of 245 wards (3 urban, 34 mixed and 208 rural). Of these, approximately 13 were excluded from the impact evaluation sample because of ineligibility for treatment (3 rural and 10 pilot wards[10]). Amongst the remaining 232 wards, the 200 largest wards were selected to form the sampling universe, based on the objective of targeting the largest potential population group. These wards have been randomly assigned to one of three groups:

T1: Local Handwashing intervention wards

T2: Local Sanitation intervention wards

T3: Local Handwashing and Sanitation interventions wards

C1: Non-intervention control wards

[9] The 200 local interventions will take place at the ward or lower level of geographic disaggregation (The Tanzanian geopolitical organization is state/district/ward). There are 2787 wards in Tanzania. Source: National Bureau of Statistics Tanzania (www.nbs.go.tz).

[10] 10 pilot wards were forcibly included into treatment based on the request of the project TTL. These 10 wards were selected based on the existence of ongoing health and water-sanitation programs. The 10 wards will receive Handwashing and Sanitation promotion (5 wards each) during the early stage of program roll out, and will likely be the first 10 wards treated in the country. Because the 10 pilot wards were selected outside of the context of the random assignment, they will not form part of the impact evaluation sample.

The final geographic clustering for the local intervention has not yet been defined. This unit may be the village, a cluster of villages, or the ward. For the purposes of sampling an initial set of treatment and control areas, the ward level analysis was conducted under the assumption that the local interventions will not spill over ward level boundaries. In a final sampling stage, wards will be sub-divided into minimum cost-efficient units of intervention, and a random sample of these units drawn for the evaluation sample and intervention. Amongst these units, a control group, C1, of approximately 50 units will be selected from the set of non-intervention wards. It is assumed that C1 has no informational spillovers given greater distances from the treatment areas.

In addition to the set of C1 “pure control” areas, a sample of non-intervention units may be drawn from within treatment wards, constituting an “internal” control group that is exposed to informational spillovers. This group, called C2 will have approximately 50 units. Under this design, C2 constitutes an “internal control” group and C1 an “external control group”. The average difference in outcomes between C2 and C1 will then give an estimate of the informational spillover effects from the local interventions. Because the extent of informational spill-overs and potential impacts are uncertain, this component will only be included if funding are available to collect data on an additional group of 50 units, and the interventions are targeted to a geographical unit below the Ward.

Local HW and TSSM interventions will be randomly assigned to intervention units within treatment wards. Local treatments are currently defined as treatment “units,” and will likely comprise a collection of hamlets or villages, in accordance with optimal minimum cost-efficient unit of intervention, given the nature of the local intervention design. For the purposes of the evaluation design, treatment units must be confined geographically to a ward, that is, they can not spill over a ward boundary. All interventions that spill across ward boundaries would be classified as regional, and not considered under the evaluation design for the local component. The final treatment sample to be included in the impact evaluation sample will be composed of the following groups:

**Table 1: Overview of Treatment and control groups**

| T/C | Group | Intervention | Number |
|---|---|---|---|
| T1 | Treatment 1 | Social marketing, local mass media and direct consumer contact for Handwashing | 47 |
| T2 | Treatment 2 | Social marketing, local mass media and direct consumer contact for Sanitation (TSSM) | 47 |
| T3 | Treatment 1+2 | Social marketing, local mass media and direct consumer contact for Handwashing and Sanitation (TSSM) | 48 |
| C1 | Control 1 | Non-intervention sites within non-treatment Wards (External Controls) | 50 |
| C2[11] | Control 2 | Non-intervention sites within treatment Wards (Internal Controls) | 50 |
| | | Total | 242 |

The impact evaluation analysis will estimate the causal impact of the HW and TSSM interventions by comparing the average outcomes in treatment and comparison areas. The following comparisons will yield the average treatment effects (estimated impacts) of the program on primary outcome indicators.

**Table 2: Estimated Impacts:**

| Comparison of outcomes | Interpretation of Impact Analysis |
|---|---|
| T1 – C1 | Impact of Social marketing, local mass media and direct consumer contact for Handwashing |
| T2 – C1 | Impact of Social marketing, local mass media and direct consumer contact for Sanitation (TSSM) |
| T3 – C1 | Impact of Social marketing, local mass media and direct consumer contact for Handwashing and Sanitation (TSSM) |
| T3- (T2+T1)/2 | Additive effects of HW & TSSM |
| C2-C1 | Informational Spill-Overs |

It is important to note that within treatment districts, local and regional interventions will be conducted simultaneously. Since all wards within a treatment district will presumably be exposed to the regional interventions, the analysis proposed here will estimate the marginal effect of local interventions, that is, the effect of localized interventions net of the impact of regional interventions.

[11] Control group C2 will be included subject to funding availability and depending on the geographical unit of implementation.

## C. Promotion of HW and TSSM at the regional level: Quasi-experimental designs

The local HW and TSSM interventions will be conducted jointly with regional media campaigns, for example using radio advertisements to promote handwashing and sanitation. The units of intervention of the regional interventions will be a cluster of wards or districts that form a "natural" area of mass media influence, such as the area of influence for a radio station or newspaper. Regional level interventions are ultimately expected to cover all areas with local level interventions, meaning that at most 10 "units" or regional intervention (10 districts) would be covered[12]. As such, the number of units of intervention for regional level interventions is expected to be too small for a purely randomized strategy. Two quasi-experimental approaches are proposed: (1) matched-pairs of districts on the ten treatment areas, randomly phased in to early and late treatment groups. Under this strategy, measurement of impacts will use primary data collected for the local interventions, but only short run impacts, such as those collected in the longitudinal diarrhea monitoring survey will be available for impact analysis (thereafter any comparison would estimate differential exposure to treatment). (2) A matched difference in difference strategy using existing data sources. Under this strategy, treatment areas would be matched to non-treatment areas. Existing data will be considered to verify the feasibility of this strategy, based on the existence of comparable outcome indicators and the likelihood of follow-up data collection within the period required for production of impact analyses.

While the feasibility of strategy (1), using primary data collection, is subject o confirmation by program operations, it is considered a viable strategy under the following conditions:

i. Districts can be randomly assigned to early and later treatment phases based on matched pairs.
ii. A minimum time period (for example six months) exist between commencement of regional media campaigns in the early treatment districts and late treatment districts.

Under these conditions, the short-run impacts of the regional media campaigns will be estimated comparing the control groups (C1) of early treatment districts with the average outcomes of the late-treatment districts. Improved balance on the sample can be achieved by matching households based on baseline characteristics, and differencing out pre-existing differences. Longitudinal diarrhea monitoring data would likely be the primary source for the impact analysis, given that a full evaluation sample follow-up survey would not take place until after all treatment districts had received a minimum amount of exposure to treatment.

[12] The units of regional intervention will be fewer than 10 for example if more than one treatment district fall within the coverage radius of the same region.

## V. COSTING[13]

Cost-benefit and cost-effectiveness and analyses of the HW, TSSM, and combined interventions are a central part of this evaluation. The goal of these analyses is to inform future programming and policy by demonstrating the allocative and technical efficiency, respectively of each intervention. Both financial and external costs, program effectiveness, and benefits will be assessed for a period of one year of intervention. Cost-effectiveness will be calculated by comparing the total costs versus the number of healthy years gained (DALYs prevented). Similar analyses will estimate the costs of and effects on productive time lost caring for sick children, and potential long-term income benefits of reduced stunting and cognitive development. Final cost-effectiveness ratios will be calculated in US dollar per healthy years gained (total costs/health effects) and compared within and across the three interventions. Cost-effectiveness ratios will also be presented for households, provider, and social perspectives (Borghi et al. 2002). Cost-benefit ratios will combine the imputed economic value of all the benefits and compare them with the full economic costs of the interventions.

Cost-effectiveness and cost-benefit ratios will be presented on a total, average, and per capita basis for one year periods, will be annualized for length of life calculations (using WHO assumptions), and will be projected based on estimated population growth over an appropriate period. These will also be disaggregated to identify the differential benefits by economic, social and demographic sub-populations.

### I. Analysis of Costs

Costs, including direct program costs and other costs that may be incurred by the community, facilities, and the target population as a result of their intervention participation, will be assessed. Total costs (the sum of all costs required to set up, implement, and sustain the intervention), average costs, and cost per capita (total costs per year divided by the total number of target population members) will be calculated. Table IV presents an overview of the costs that will be taken into account.

[13] Costing strategy is preliminary. A fully developed costing plan is under development with the National Institute of Public Health in Mexico. Further discussions are required with project TTL regarding reporting and record keeping systems that will be used by implementing firms and government partners.

**Table IV: Costs Related to the Interventions**

| Type | Measure | Data Sources |
|---|---|---|
| **Direct Costs to Program Provider**[14] | Total cost per intervention including:<br>• Investment Costs (construction, vehicle, equipment)<br>• Recurrent Costs (personnel, training, supplies, communication, transport, utilities, etc.)<br>• Maintenance costs | WSP program records (expenditure statements, budgets)<br>Routine Activity Reports from Social Marketing Firms (documenting program outputs and inputs, supervision, monitoring activities, etc.) |
| **Indirect Costs to Program Provider** | Total time lost by volunteers (e.g., teachers, community members)<br><br>Total additional donated output | Routine Activity Reports from Social Marketing Firms (documenting program outputs and inputs on the part of volunteers)<br><br>Records of any additional program input |
| **Direct Cost to Households** | Total cost (monetary or otherwise) incurred in purchasing necessary intervention components (water, soap, latrines, latrine maintenance) | Self-report (baseline and follow-up questionnaire) |
| **Indirect Costs to Households** | Work time lost to household from participating in intervention or intervention-related tasks | Self-report (baseline and follow-up questionnaire) |
| **Direct Cost to Facility** | Total costs associated with facility personnel's participation in intervention activities | Self-report of total number of clinic/hospital visits (baseline and follow-up)<br><br>Health Management Information Systems (TBD) |
| **Indirect Cost to Facility** | Health worker days lost to diarrheal disease | Health Management Information Systems (TBD) |
| **Direct Cost to Society** | Expenditure associated with burden of disease | Estimations based on Government Budgets, WSP and/or WHO. (TBD) |

[14] The program provider is considered to be the Government of Tanzania.

| Type | Measure | Data Sources |
|---|---|---|
| **Indirect Costs to Society** | Productivity costs associated with burden of disease and death<br>Productive worker days lost<br>Productive school days lost due to illness<br>Child days lost due to illness | WHO estimations (TBD) |

## II. Analysis of Effectiveness

The impact evaluation will assess the effectiveness of each intervention through the impact evaluation. Potential measures of effectiveness include behavioral outcomes such as the number of targeted households (mothers, caregivers) that changed behavior, the average increase in handwashing with soap and/or sanitation-related behaviors as well as health, development and economic outcomes. One of the primary outcomes of interest in the context of this intervention is diarrheal disease (WHO, 2004); the effectiveness of each intervention will be the impact on incidence of diarrheal disease among our target population, and the development and economic impacts these trigger. Number of healthy years gained (DALYs prevented) per capita may will be calculated from this measure.

## III. Analysis of Benefits

The measurement of benefits associated with each intervention will be computed for each beneficiary group based on information collected from households and facilities. Societal benefits will be considered as the sum of household and facility savings (Borghi, 2002). Table V presents an overview of measures of effectiveness to be used in this study.

**Table V: Benefits Related to the Interventions**

| Type | Input | Estimation Method |
|---|---|---|
| **Direct Household Benefits** | Money and time saved from averted health care consultations, treatment, medication, opportunity costs (time traveling to facility), transport costs, and other costs associated with health care visit (e.g., food, drink), | Average cost per medical consultation (including opportunity, transport, and other costs) Multiplied by number of cases averted |
| **Indirect Household Benefits** | Reduced funeral Costs | Average cost per funeral multiplied by number of cases averted |
| | Reduced lost income from caregiver days<br>Reduced number of school days missed | Average cost per day lost to work multiples the number of cases averted |
| | Decreased productivity loss from child death | |
| **Direct Facility Benefits** | Resources saved from averted medical visits and hospitalizations (multiplying health service unit cost times the number of cases averted) | HMIS questionnaire and WHO regional costs databases |
| **Indirect Facility Benefits** | Fewer workers falling sick | HMIS questionnaire and WHO regional costs databases |
| **Direct Societal Benefits** | Less expenditure on treatment of citizens with diarrheal diseases? | HMIS questionnaire and WHO regional costs databases |
| **Direct Societal Benefits** | Less productivity loss associated with burden of disease and death<br>Fewer productive worker days lost<br>Fewer productive school days lost due to illness<br>Fewer child days lost due to illness | HMIS questionnaire and WHO regional costs databases |

## IV. Sensitivity Analysis

Cost benefit analysis will include standard sensitivty analysis  to tests the sensitivity and reliability of the results. Sensitivity analysis identifies those input parameters that have the greatest influence on the outcome, repeats the analysis with different input parameter values, and evaluates the results to determine which, if any, input parameters are sensitive. If a relatively small change in the value of an input parameter changes the alternative selected, then the analysis is considered to be sensitive to that parameter. If the value of a parameter has to be doubled before there is a change in the selected alternative, the analysis is not considered to be sensitive to that parameter. The estimates for sensitive input parameters should be re-examined to ensure that they are as accurate as possible

## VI. SAMPLE DESIGN and RANDOM ASSIGNMENT OF TREATMENT

The primary objective of the HW and TSSM promotion interventions is to improve the health and welfare of young children. As such a household level sample is proposed to capture a minimum effect size of 20% on the key outcome indicator of diarrhea prevalence amongst children ages 0-24 months at baseline (approximately 15-39 months old by first follow-up, depending on agreed timeline for the follow-up survey). The decision to sample households with children in this age group was done under the assumption that health outcome measurements for young children in this age range are most sensitive to changes in hygiene in the environment. Data will be collected for household members of all age ranges and corresponding impact analysis will be conducted for older children and adults as well. Given this construct, it is important to note that the sample is representative only of households with 0-24 month old children in the 200 treatment Wards, and all associated power calculations are made in reference to this group. The sample is designed with the primary objective of producing internally valid estimates of program impacts, and will not necessarily be suitable for computing country or district level population statistics without appropriate corrections.

The final sample for the evaluation sample will consist of approximately 3500 households with children between 0 and 24 months of age at baseline. The sampling process includes four primary stages:

### A. Sample Selection Stage 1: District Selection and Random Assignment at District Level (regional)

A set of 10 districts from across Tanzania have been strategically selected a priori to receive the HW and TSSM interventions. These districts are: Igunga, Iringa, Karagwe, Kiteto, Kondoa, Masasi, Mpwapwa, Musoma, Rufiji and Sumbawanga (see Appendix A). The 10 districts are geographically diverse, covering districts spread throughout the country in an effort to reflect the geographic diversity of the country. Although the sample is not fully representative at a national level by construction, the geographic diversity should help ensure that the impacts measured in this sample are broadly indictative of the impacts that can be expected in a national program. But the targeted districts will surely differ in some dimensions from the local conditions of the remaining 119 districts in country. In particular, HVC districts and Water/Sanitation districts may have self selected into treatment. While this is not a threat to the internal validity of the experiment, we will explore the implications for its external validity. Further analysis will be done to examine the presence of observable differences between the treatment and comparison districts that could influence the effectiveness of the interventions when applied nationally.

**Table VI: Treatment Districts & Sanitation Gap**

| | District | Population | Unserved | % Unserved |
|---|---|---|---|---|
| **1.** | Igunga | 324,094 | 302,306 | 93% |
| **2.** | Iringa | 245,623 | 232,802 | 95% |
| **3.** | Karagwe | 424,287 | 406,488 | 96% |
| **4.** | Kiteto | 152,269 | 130,645 | 86% |
| **5.** | Kondoa | 428,090 | 173,912 | 41% |
| **6.** | Masasi | 440,987 | 364,623 | 83% |
| **7.** | Mpwapwa | 253,602 | 39,364 | 16% |
| **8.** | Musoma | 329,824 | 315,405 | 96% |
| **9.** | Rufiji | 202,001 | 157,373 | 78% |
| **10.** | Sumbawanga | 371,749 | 351,094 | 94% |
| | **Total** | **3,172,526** | **2,474,012** | **78%** |

For implementation of the regional level impact evaluation using primary data, we randomly assign districts into an early (phase 1) and later (phase 2) treatment groups based on matched pairs (matched on population size). The actual timing between phases will be an operational decision, based on capacity to implement. However, it is assumed that approximately 6 months of differential exposure to treatment would be the minimum necessary to warrant this approach. The feasibility of this or similar approaches will be discussed with the implementing agency and the project TTLs, prior to confirming its validity as an impact evaluation strategy for the regional interventions. Districts are assigned to the following treatment phases:

**Table III: Treatment Phase**

| District | Population | Random_Number | Treatment Phase |
|---|---|---|---|
| Karagwe | 425476 | 2.789891 | 1 |
| Kondoa | 429824 | -0.192865 | 2 |
| Sumbawanga Rural | 373080 | 0.8707055 | 1 |
| Masasi | 408401 | -1.593113 | 2 |
| Igunga | 325547 | 1.101791 | 1 |
| Musoma Rural | 330953 | -0.6407495 | 2 |
| Iringa Rural | 245623 | 0.8697362 | 1 |
| Mpwapwa | 254500 | 0.2989207 | 2 |
| Kiteto | 148200 | -0.021485 | 1 |
| Rufiji | 196369 | -0.7791277 | 2 |

Thus, under this design, Karagwe, Sumbawanga Rural, Igunga, Iringa Rural and Kiteto districts will be the first 5 districts to implement the regional interventions (presumably in conjunction with local interventions), followed by the remaining five districts (Kondoa, Masasi, Musoma Rural, Mpwapwa and Rufiji).

### B. Sample Selection Stage 2: Ward Selection and Random Assignment of Treatment to Wards (local):

Data used for sampling at the ward level is 2002 census data from the National Bureau of Statistics. There are a total of 245 wards in the 10 treatment districts. A sample of 220 wards have been selected as potential intervention sites by matching wards into groups of four within districts based on population size, and randomly assigning each to one of four groups, three treatment conditions and one non-treatment control group. A total of 142 wards are assigned to treatment and 48 wards assigned to control, with the remainder "wait listed". The remaining wards are not part of the evaluation sample, although 10 wards with HVC and water/sanitation villages will receive treatment in the intervention design phase. The sampling framework of 220 wards was selected through the following procedure[15]:

1. Exclude urban wards (3): Of the 245 wards in 10 districts selected in Stage 1, approximately 99% (208) are identified in the census data as rural wards, 14% (34) as mixed, and 1% (3) as urban. Given that the intervention is targeted at rural areas, the 3 urban wards are excluded from the sampling universe.

2. Exclude HVC and pilot water/sanitation wards: The MOH and MOW have implemented a number of pilot health and sanitation activities in a total of 10 villages which are to be forcibly included in the handwashing and sanitation promotion interventions (5 villages each). Given that these villages are forcibly included in program treatment, 5 wards containing MoH "Healthy Village Campaign" villages and 5 wards with MOW sanitation villages will be excluded from the impact evaluation sample[16]. Additionally, the handwashing and sanitation intervention pilots will take place in these 10 wards during the initial phase of planning and design of the intervention.

3. Smallest wards that form a group of three or fewer within a district: The remaining 232 wards were matched on population size within districts to form groups containing four wards each. Given the objective of targeting the largest wards in the sample to reach the largest possible number of beneficiaries (and arguably the most accessible areas), the set of smallest wards, based on population size, containing 3 or fewer wards were dropped from the sample. In total, 12 of the smallest wards were dropped, for a sampling universe of 220 wards, matched into sets of 4 wards.

4. Random assignment to treatment: within each group of 4, wards are randomly assigned to one of the following treatment groups[17]:

---

[15] See Appendix B for sampling code in STATA.

[16] When selecting the sample, the country TTL instructed that the 5 pilot sanitation wards should be selected randomly from amongst the 49 wards with ongoing sanitation activities (rwss) in the sample. The 5 health village campaign wards were identified and excluded from the sample.

[17] Random numbers were assigned to each ward within a pair of four, and then sorted based on the random number.

(T1): Handwashing only
(T2): Sanitation only
(T3): Handwashing and Sanitation
(C1): Control

Appendix C presents the list of Wards, randomly assigned to each of the treatment and control groups. There is good balance on available population characteristics, including population (total, male, female), households and household size, and proportion rural (results available upon request). 190 wards have been assigned to the initial treatment/comparison groups. All remaining wards are maintained on replacement lists, in case one of the original wards drops from the sample for operational reasons (for example the ward is inaccessible or the intervention is refused). There are between 7 to 8 replacement or substitute wards for each group. The number of substitutes to include in the final sample entails budget implications, and so will be determined upon consultations with the Global program and country TTLs. If the firms implementing the intervention are able to confirm the 142 treatment areas in the evaluation sample before the baseline, then including additional replacements in the sample will not be necessary. If this is not possible to determine the feasibility of implementation ex-ante, then the sample would include between 0 and 30 additional replacement wards.

### C. Sample Selection Stage 3: Household cluster selection

Final clustering of households in the sample will depend on the unit of intervention selected. The unit of intervention will be selected as the minimum cost efficient unit of intervention. Units of intervention must include the totality of a well defined area, be that locality, community or enumeration area that constitutes a “natural” area of intervention within the context of the HW and TSSM interventions. Units of intervention must be confined to a ward, and not spill over into adjacent wards. Although units containing MoHWS “Healthy Village Campaign” villages will be included in the treatment sample, but will be excluded from the evaluation sample (see Stage 2).

### D. Sample Selection Stage 4: Household selection:

Within the set of clusters identified in stage 3, a random sample of 17 households (to be confirmed) containing at least one child between 0-24 months of age will be drawn. Sample selection procedures at this stage will be designed by the survey firm, with approval by the principal investigators. Complete questionnaires will be collected on all households included in the sample.

## E. Power Calculations

The primary objective of the impact evaluation is to estimate the causal impact of the program, that is, to detect a statistically significant difference in the mean outcomes of the treatment and control groups. Power calculations are important to determine the optimal sample structure required to detect a pre-determined effect size. Desired power for a study is typically set at 0.8 or 0.9, meaning that there is an 80 or 90 percent probability that the desired effect size will be captured in the analysis. For the purposes of this study, a power of 0.8 (the probability of correctly determining there is a program effect when there is one) and significance of 0.05 (the probability of falsely concluding there is a program effect when in reality there is none) will be set as the minimum acceptable power and significance, respectively.

In the Tanzanian HW and TSSM case, there are a total of 95 HW and 95 TSSM units of intervention available for the sample, clustered into three groups: 47 HW clusters, 47 TSSM clusters and 48 HW & TSSM clusters. Each of these units also represents a sampling cluster, with numerous households treated within each cluster. If households within a cluster tend to share outcomes that are common within a cluster, then each additional household sampled within a cluster will add less information to the analysis. Thus, the effect size that can be detected by the analysis is largely driven by the number of clusters (or units of intervention) in the evaluation sample, and relatively less by the number of households that are observed within each cluster. Taking this into consideration, Galiani {insert references} has estimated key parameters from Luby et al {insert references} in Pakistan. Using the Luby data, Galiani proposes a mean diarrhea prevalence of 0.086 and intra-cluster correlation of 0.105[18]. Assuming perfect compliance, a minimum cluster size per study arm of J = 47 and a minimum desired detectable effect size of 15% and 20% (standardized effect size equal to 0.32 and 0.44, respectively[19]), we estimate the sample required per cluster as:

Table VII. Sample sizes per cluster - simulation

| | Effect size = 15% | Effect size = 20% |
|---|---|---|
| Power = 0.8 | 15 | 5 |
| Power = 0.9 | 49 | 9 |

Drawing from Galiani's estimates taking into account low compliance levels (0.50) and a desired detectable effect size of 20%, it is estimated that each arm of the study will require a minimum of 45 groups and 17 households per group are required to achieve a power of 0.8. Taking these into consideration, two sample structures are proposed.

[18] Variance of the individual effect = 0.001662 and variance of the group effect = 0.000174. The intra-cluster correlation, $\rho = 0.000174/0.001662 = 0.105$.

[19] The standardized effect size is calculated as the difference in mean outcome between the treatment and control groups divided by the standard error of the outcome, given here by the square root of the variance of the variance of the individual effect. Standardized effect size for 15% reduction in diarrhea from a mean prevalence of 0.086 in the control group requires a mean prevalence in the treatment group of 0.073, which translates into a standardized effect size of ($0.0129/\sqrt{(0.001662)} = 0.32$). Standardized effect size for 20% reduction in diarrhea from a mean prevalence of 0.086 in the control group requires a mean prevalence in the treatment group of 0.068, which translates into a standardized effect size of ($0.018/\sqrt{(0.001662)} = 0.44$)

Sampling option number 1 includes a minimum of 47 clusters per study arm with 4 arms, and 17 households per cluster. Sampling option number 3 includes a minimum of 47 clusters per study arm with 5 arms and 13 households per cluster. Under sampling option 1, a minimum effect of 15.5% (standardized effect size of 0.33) is detectable with power 0.8. Under option 2, a minimum effect of 16.5% (standardized effect size of 0.35) is detectable with power 0.8.

**Table VIII: Proposed Sample Structures**

| | Number of Clusters | Number of households<br>Sample Option 1: 17 HHs per cluster | Number of households<br>Sample Option 2: 13 HHs per cluster |
|---|---|---|---|
| Treatment Group 1 (T1): Local Handwashing | 47 | 799 | 611 |
| Treatment Group 2 (T2): Local Sanitation | 47 | 799 | 611 |
| Treatment Group 3 (T3): Local Handwashing and Local Sanitation | 48 | 816 | 624 |
| Control Group 1 (C1): External Controls ("pure controls") | 48 | 816 | 624 |
| Control Group 2 (C2): Internal Controls ("exposed controls") | 48 | NA | 624 |
| Total Households (lower bound - no substitutes) | - - | 3230 | 3094 |
| Total Households (upper bound - all substitutes) | | 3740 | 3484 |

Note: Total of 100 HW clusters and 100 TSSM intervention clusters. 10 pilot clusters in HVC and sanitation villages (5HW, 5TSSM) are excluded from the evaluation sample, for a total evaluation sample of 190 clusters, divided into groups of 47 HW, 47TSSM and 48 Crossover. The final sample size will be a function of the number of substitute wards included in the sample.

**Table IX: Sampling Flowchart (sampling option 1)**

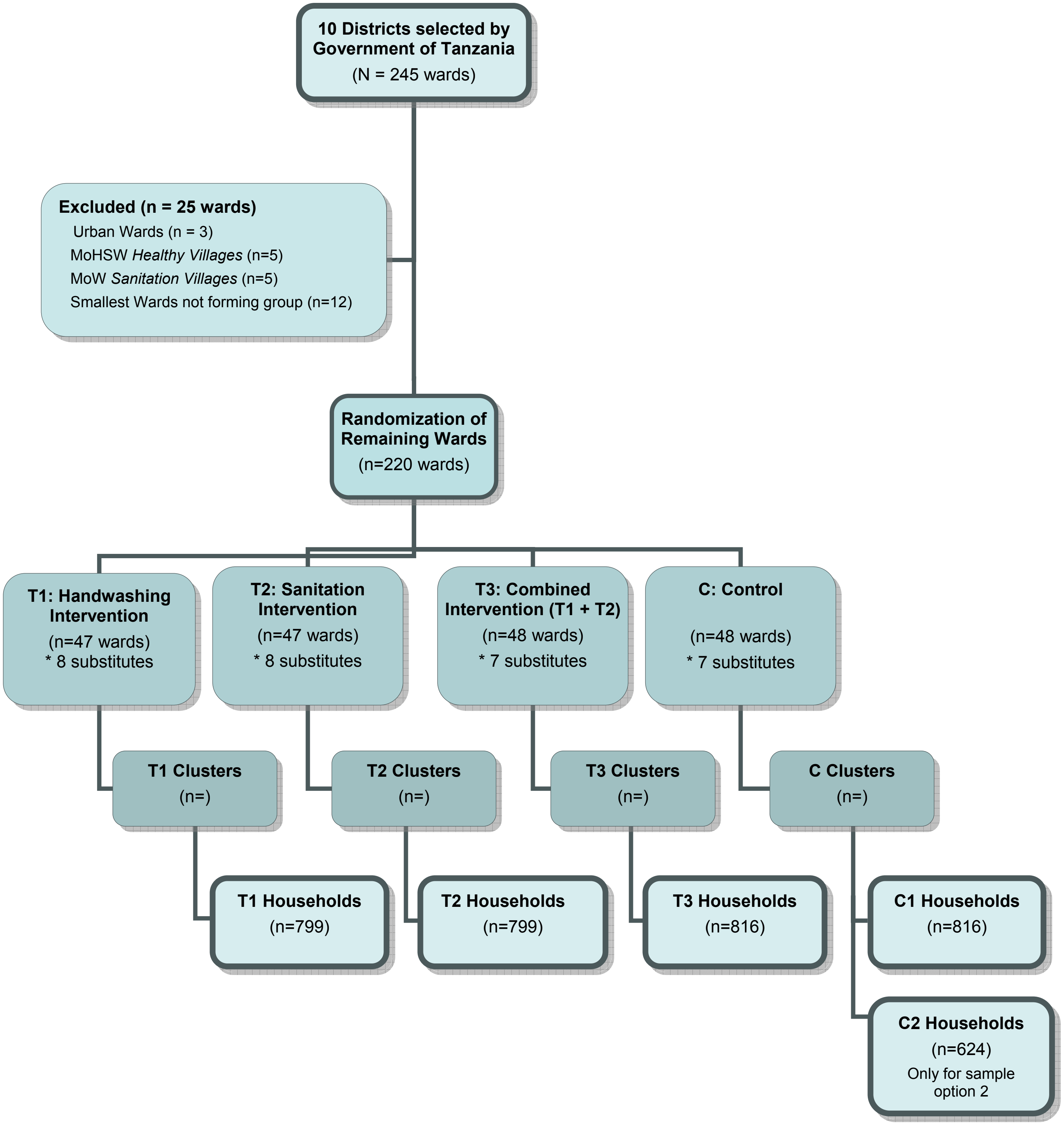

## VII. SURVEY INSTRUMENT and ROUTINE MONITORING DATA

The base survey instruments and routine monitoring data collection protocols are under development by the global program. The Tanzania impact evaluation team will work with the survey firm to adapt the survey instrument to the local context, and introduce additional country specific questions, modules and protocols, as needed.

## VIII. IMPACT EVALUATION TIMELINE

Coordinating the timing of the intervention implementation with baseline and follow-up surveys is critical for both the project operations and the IE. The intervention design piloting is proposed to begin at the earliest possible date in the 10 pilot wards selected for treatment (but excluded from the evaluation sample). Initiating the intervention in a set of non-intervention wards is important from the standpoint of the evaluation, since this experience will allow the implementing agency to test, improve and standardize its approach to handwashing and sanitation promotion, and based on this experience scale up with an intervention that is both well formulated and documented. Simultaneously, the baseline survey contracting and pilot testing will take place (possibly in some of the pilot areas), with the objective of fielding the full baseline survey in the first five districts (district group 1) by March 2008. Assuming approximately 6-8 weeks of survey work to complete the sample in these districts, the intervention may commence by May 2008 in district group 1, following completion of the baseline survey. Note that to avoid changing behavior through expectations it is preferable that the intervention is not announced in a ward until AFTER the baseline has been completed. Survey work would commence in the second group of 5 districts (district group 2) immediately following completion of district group 1, and finalizing the full sample by June 2008. The intervention could then roll into district group 2 areas following the conclusion of the survey. If the regional impact evaluation using primary data is feasible, we suggest a minimum 6 month lag between the introduction of regional level treatments between district groups 1 and 2. Alternative roll-out schedules for the implementation, which are also amenable to the regional evaluation, can be considered (for example a random phase in by district).

It is expected that data capturing will take place on a rolling basis as the survey is implemented, whether using computer assisted survey technology, or through paper and pencil surveys which are captured in the field or sent immediately for capturing at a central station. This will allow for ongoing data checks to measure the accuracy and consistency of the surveys, as they are collected, and to correct any irregularities that are detected in real time. With the expectation that baseline data will be available for analysis starting in July 2008, the final baseline analysis and data are expected by September, 2008. We present a detailed timetable below.

**Tentative Timeline for Baseline Data Collection and Intervention Roll Out**

| Date | Operations | Impact Evaluation |
|---|---|---|
| Jan 08-Feb 08 | **Contracting of firms and intervention design** | **Contracting, piloting and preparations** |
| March 08-April 08 | Piloting of intervention activities in areas confined outside of the evaluation sample (10 pilot wards) | Baseline data collection – first batch of 5 districts |
| May 08- June 08 | Intervention begins in first batch of 5 districts (begin activities in surveyed Wards) | Baseline data collection –second batch of 5 districts |
| July 08-Dec 08 | Earliest date for intervention begins in second batch of districts (begin activities in surveyed Wards) | Intervention in first batch of 5 districts |

## Table X: Tanzania HW & TSSM Impact Evaluation Timeline (subject to change)

| | 2007 | | | | | | | 2008 | | | | | | | | | | | | 2009 | | | | | | | | | | | | 2010 | | | | | | | | | | |
|---|---|---|---|---|---|---|---|---|---|---|---|---|---|---|---|---|---|---|---|---|---|---|---|---|---|---|---|---|---|---|---|---|---|---|---|---|---|---|---|---|---|---|
| | J | J | A | S | O | N | D | J | F | M | A | M | J | J | A | S | O | N | D | J | F | M | A | M | J | J | A | S | O | N | D | J | F | M | A | M | J | J | A | S | O | N |
| Evaluation Design | X | X | X | X | X | X | X | | | | | | | | | | | | | | | | | | | | | | | | | | | | | | | | | | | |
| Site Selection | | | | X | X | X | X | | | | | | | | | | | | | | | | | | | | | | | | | | | | | | | | | | | |
| REOI | | | X | | | | | | | | | | | | | | | | | | | | | | | | | | | | | | | | | | | | | | | |
| RFP | | | | | | X | | | | | | | | | | | | | | | | | | | | | | | | | | | | | | | | | | | | |
| Contracting of Survey Firm | | | | | | | X | X | | | | | | | | | | | | | | | | | | | | | | | | | | | | | | | | | | |
| Advisory Panel | | | | X | | | | | X | X | | | | | | X | | | | | | | | | | | | X | | | | | | | | | | | | X | | |
| Human Subjects (NIMR) | | | | | | | | X | X | | | | | | | | | | | | | | | | | | | | | | | | | | | | | | | | | |
| Survey Instrument pre-pilot & translation | | | | | | | | X | X | | | | | | | | | | | | | | | | | | | | | | | | | | | | | | | | | |
| Field Training and Pilot Testing | | | | | | | | | X | X | | | | | | | | | | | | | | | | | | | | | | | | | | | | | | | | |
| Intervention Pilot in Water and HVC villages | | | | | | | | | | X | X | X | | | | | | | | | | | | | | | | | | | | | | | | | | | | | | |
| Baseline Survey District Group 1 | | | | | | | | | | X | X | | | | | | | | | | | | | | | | | | | | | | | | | | | | | | | |
| Intervention Roll out in District Group 1 | | | | | | | | | | | | X | X | X | X | X | X | X | X | X | X | X | X | | | | | | | | | | | | | | | | | | | |
| Baseline Survey District Group 2 | | | | | | | | | | | | X | X | | | | | | | | | | | | | | | | | | | | | | | | | | | | | |
| Intervention Roll out in District Group 2 | | | | | | | | | | | | | | | | | | | X | X | X | X | X | X | X | X | X | X | X | X | | | | | | | | | | | | |
| Data capturing/ processing | | | | | | | | | | X | X | X | X | | | | | | | | | | | | | | | | | | | | | | | | | | | | | |
| Final baseline data and report from Survey firm | | | | | | | | | | | | | | X | | | | | | | | | | | | | | | | | | | | | | | | | | | | |
| Baseline Data analysis | | | | | | | | | | | | | | X | X | | | | | | | | | | | | | | | | | | | | | | | | | | | |
| Preliminary baseline report | | | | | | | | | | | | | | | X | | | | | | | | | | | | | | | | | | | | | | | | | | | |
| Baseline report review and revision | | | | | | | | | | | | | | | | X | | | | | | | | | | | | | | | | | | | | | | | | | | |
| Final baseline report | | | | | | | | | | | | | | | | X | | | | | | | | | | | | | | | | | | | | | | | | | | |
| Contracting of Survey Firm | | | | | | | | | | | | | | | | | | | | | | | | | | | | | X | X | X | | | | | | | | | | | |
| Survey Instrument pre-pilot | | | | | | | | | | | | | | | | | | | | | | | | | | | | | | | X | | | | | | | | | | | |
| Field Training and Pilot Testing | | | | | | | | | | | | | | | | | | | | | | | | | | | | | | | | X | | | | | | | | | | |
| End-line survey | | | | | | | | | | | | | | | | | | | | | | | | | | | | | | | | | X | X | X | X | | | | | | |
| Data capturing/ processing | | | | | | | | | | | | | | | | | | | | | | | | | | | | | | | | | | | | X | X | | | | | |
| Data analysis | | | | | | | | | | | | | | | | | | | | | | | | | | | | | | | | | | | | | X | X | | | | |
| Preliminary End-line evaluation report | | | | | | | | | | | | | | | | | | | | | | | | | | | | | | | | | | | | | | | X | | | |
| End-line evaluation report review | | | | | | | | | | | | | | | | | | | | | | | | | | | | | | | | | | | | | | | | X | | |
| Final evaluation report | | | | | | | | | | | | | | | | | | | | | | | | | | | | | | | | | | | | | | | | X | | |

**Timing of follow up survey under discussion.**

**APPENDIX A**

**10 Intervention Districts Selected by the Government of Tanzania**

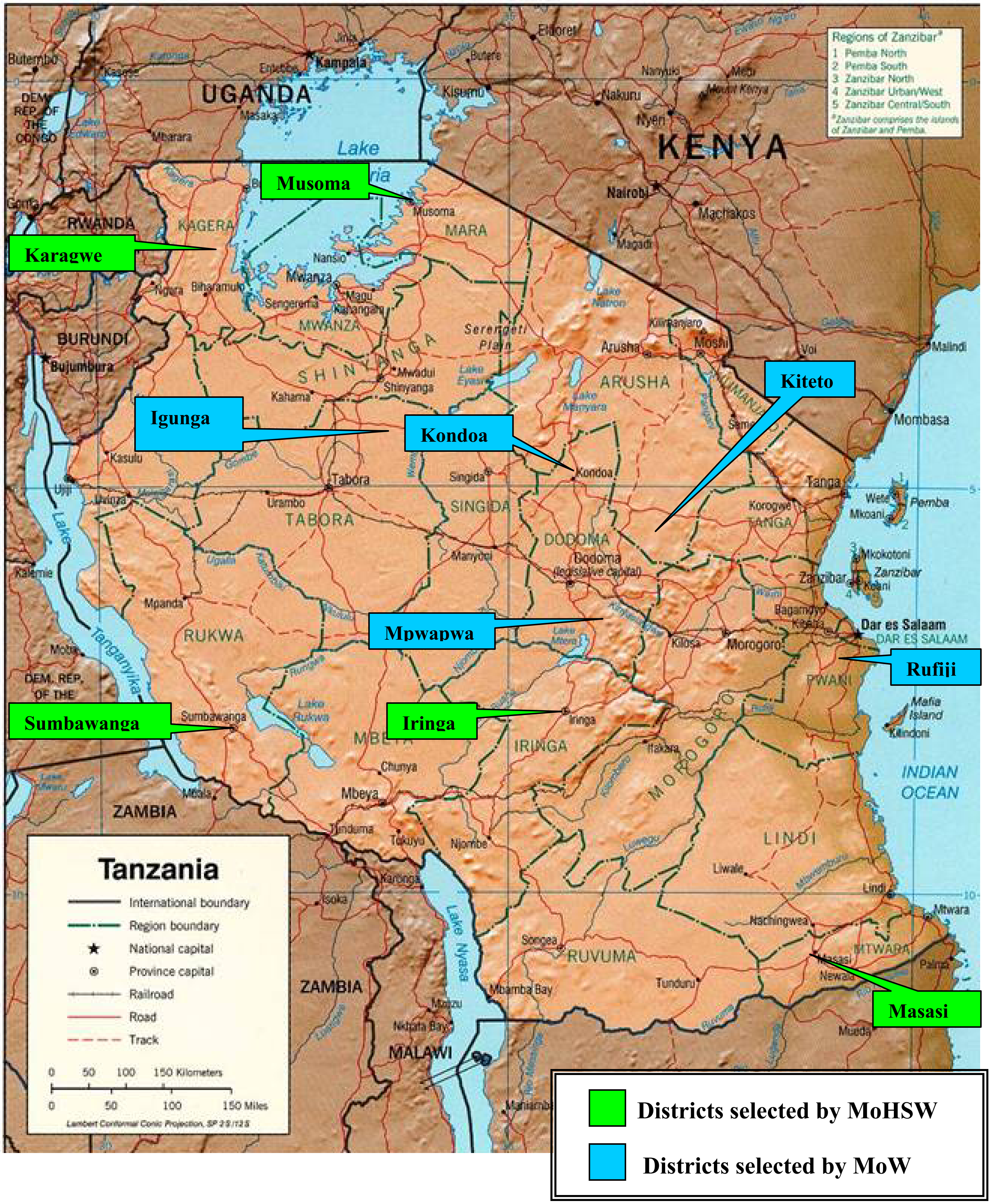

## Appendix B: Ward sampling and random assignment of treatment

## STATA CODE

```
#delimit;

clear;
cap log close;
set more off;
set mem 50m;
set matsize 100;
program drop _all;
*==========================*
September 14, 2007
Code by S Martinez
*==========================*;

*==========================*;
*temporary files;
tempfile temp1 temp2 temp3;
*==========================*;

*=========================*;
use "C:\Documents and Settings\wb276487\My
Documents\Wb\Projects\Tanzania\HandWash\Sampling\Baseline\2002 Census - Ward
level\HW_TSSM_Wards2.dta", clear;

*generate unique state and district IDs;
egen state_id = group(state);
egen district_id = group(state district);
gen rural = type == "Rural";

*Drop Urban Wards;
drop if type == "Urban";

*Drop 10 wards with Healthy Village Campaigns (these must forcibly be included
in the treatment group);
*draw a random sample of 5 Sanitation (RWSS) wards for pilot;
set seed 111007;
gen random_rwss = invnormal(uniform()) if rwss ==1;
sort rwss random_rwss;
gen n =1;
gen count = sum(n) if rwss==1;
gen pilot_ward= 1 if (count >=1 & count <=5) |  handwashing_pilot_5 ==1;
gen sanitation_pilot_5 = 1 if (count >=1 & count <=5);
drop n;
drop count;

browse if handwashing_pilot_5 ==1;
browse if sanitation_pilot_5 ==1;
drop if pilot_ward ==1;

*********************
*Sort population by district;
*Note that sorting highest to lowest total population, we will exclude the
smallest wards from
forming sets of 4 wards. Countrh TTLs Nathan P. and Ousseynou D. have expressed
that for operational reasons, it is desireable to eliminate the smallest wards
from the sampling framework for two primary reasons, first to reach a larger
target population with the intervention, and second becuase smaller wards are
likely less accessible;

gsort state_id district_id -total;
```

```
gen n =1;
bys  state_id district_id: gen count = sum(n);
gen count_temp = count/4;
gen set = 0 if count_temp>0 & count_temp <=1;
foreach x of numlist 1/10{;
replace set = `x' if count_temp >`x' & count_temp <=(`x'+1);
};

*Assign random number to all wards;
set seed 091407;
bys state_id district_id set: gen random_number = invnormal(uniform());
gsort state_id district_id set -random_number;
bys state_id district_id set: gen random_ward = sum(n);

label var random_ward "random assignment T=1,2,3,4";

*drop wards that do not fall within a set of 4;
bys state_id district_id set: egen max_set = max(random_ward);
drop if max_set <=3;

*select random sample of 47 HW only, 47 TSSM only, 48 HW+TSSM and 48 internal
controls. Remaining go to replacement list;
gsort random_ward -random_number;
bys  random_ward: gen count_replace = sum(n);

gen treatment_handwashing = random_ward ==1 & count_replace <=47;
gen replacement_handwashing = random_ward ==1 & count_replace >=47;
bys district set: egen max_treat_hw = max(treatment_handwashing);

gen treatment_sanitation = random_ward ==2 & max_treat_hw ==1;
gen replacement_sanitation = random_ward ==2 & max_treat_hw ==0;

gen treatment_hws_temp = 1 if random_ward ==1 & count_replace ==48;
bys district set: egen max_treat_hwandsan = max(treatment_hws_temp);
gen treatment_HWandSanitation = 1 if random_ward ==3 & (max_treat_hwandsan ==1
| max_treat_hw ==1);
replace treatment_HWandSanitation = 0 if treatment_HWandSanitation==.;
gen replacement_HWandSanitation = 1 if random_ward ==3 &
treatment_HWandSanitation ==0;

gen control = random_ward ==4 & (max_treat_hwandsan ==1 | max_treat_hw ==1);
replace control = 0 if control ==.;
gen replacement_control = 1 if random_ward ==4 & control ==0;

label var treatment_handwashing "Handwashing only treatment wards = 1";
label var treatment_sanitation  "Sanitation only treatment wards = 1";
label var treatment_HWandSanitation "Handwashing and Sanitation treatment wards
= 1";
label var control "Control wards (no interventions) = 1";

preserve;

keep  state district wardno ward type male female total numberhhs averagesize
healthyvillage
rwss handwashing_pilot_5 state_id district_id rural sanitation_pilot_5 set
random_number
random_ward treatment_handwashing replacement_handwashing treatment_sanitation
replacement_sanitation
treatment_HWandSanitation replacement_HWandSanitation control
replacement_control;

*Browse 4 randomly assigned groups;
*Handwashing Treatment;
```

```
browse if treatment_handwashing ==1;
*Sanitation Treatment;
browse if treatment_sanitation ==1;
*Handwashing and saniation Treatment;
browse if treatment_HWandSanitation ==1;
*Controls;
browse if control ==1;
*Handwashing Replacements;
browse if replacement_handwashing ==1;
*Sanitation Replacements;
browse if replacement_sanitation ==1;
*Handwashing and saniation Replacements;
browse if replacement_HWandSanitation ==1;
*Control Replacements;
browse if replacement_control ==1;


restore;

*Test balance on populaiton sizes amongst treatment groups;

foreach x of varlist male female total numberhhs averagesize rural {;
ttest `x' if random_ward ==1 | random_ward ==2, by(random_ward);
ttest `x' if random_ward ==1 | random_ward ==3, by(random_ward);
ttest `x' if random_ward ==1 | random_ward ==4, by(random_ward);
ttest `x' if random_ward ==2 | random_ward ==3, by(random_ward);
ttest `x' if random_ward ==2 | random_ward ==4, by(random_ward);
ttest `x' if random_ward ==3 | random_ward ==4, by(random_ward);
};
```

**AppendixC:** Random Assignment of Wards to Treatment/Control Groups

| Pilot Wards | | | | | |
|---|---|---|---|---|---|
| Handwashing Pilot Wards | | | Sanitation Pilot Wards | | |
| Region | District | Ward | Region | District | Ward |
| Dodoma | Mpwapwa | Wotta | Pwani | Rufiji | Utete |
| Rukwa | Sumbawanga Rural | Mkowe | Tabora | Igunga | Choma |
| Mara | Musoma Rural | Nyamrandirira | Dodoma | Kondoa | Bumbuta |
| Mtwara | Masasi | Namalenga | Manyara | Kiteto | Sunya |
| Iringa | Iringa Rural | Idodi | Tabora | Igunga | Nkinga |

| Intervention and Control Wards | | | | | |
|---|---|---|---|---|---|
| Region | District | Group T1 Handwashing | Group T2 Sanitation | Group T3 Sanitation and HW | Group C1 Control |
| Tabora | Igunga | Bukoko | Mbutu | Igunga | Igoweko |
| | | Mwashiku | Ziba | Naga | Itumba |
| | | Mwamashimba | Simbo | Ndembezi | Sungwizi |
| | | Nguvumoja | Igurubi | Itunduru | Kinungu |
| | | Kining'inila | Mwisi | Nyandekwa | Mwamashiga |
| | | Ngulu | Isakamaliwa | Chabutwa | Ntobo |
| Iringa | Iringa Rural | Nzihi | Ifunda | Mseke | Izazi |
| | | Nduli | Maboga | Kiwere | Wasa |
| | | Ulanda | Kalenga | Mlowa | Lumuli |
| Kagera | Karagwe | Kamuli | Kiruruma | Nkwenda | Mabira |
| | | Kimuli | Nyakakika | Kituntu | Bugomora |
| | | Kyerwa | Kaisho | Rwabwere | Igurwa |
| | | Nyakahanga | Isingiro | Kibingo | Ihanda |
| | | Kayanga | Bweranyange | Kihanga | Ndama |
| | | Kibondo | Bugene | Nyaishozi | Murongo |
| Manyara | Kiteto | Dongo | Olboloti | Partimbo | Matui |
| | | Kijungu | Dosidosi | Bwagamoyo | Makame |
| Dodoma | Kondoa | Kondoa Mjini | Goima | Chandama | Jangalo |
| | | Busi | Changaa | Bereko | Pahi |
| | | Haubi | Mrijo | Dalai | Makorongo |
| | | Kikore | Mnenia | Paranga | Kalamba |
| | | Ovada | Kisese | Suruke | Kingale |
| | | Farkwa | Kikilo | Thawi | Mondo |
| | | Kolo | Lalta | Kwadelo | Sanzawa |
| | | - | - | Soera | Kwamtoro |
| | Mpwapwa | Mpwapwa Mjini | Rudi | Chunyu | Kibakwe |
| | | Mazae | Matomondo | Kimagai | Ipera |
| | | Massa | Mbuga | Lumuma | Ving'hawe |
| Mtwara | Masasi | Mwena | Lukuledi | Nangomba | Lisekese |
| | | Chigugu | Nanganga | Likokona | Namajani |
| | | Mikangaula | Maratani | Chiungutwa | Mnavira |
| | | Marika | Sengenya | Mpindimbi | Nandete |
| | | Lumesule | Mkonona | Mkululu | Lipumburu |
| | | Mkundi | Sindano | Chiwata | Masuguru |
| Mara | Musoma Rural | Nyankanga | Buhemba | Kukirango | Etaro |
| | | Kiriba | Butiama | Bwasi | Nyakatende |

| | | | | | |
|---|---|---|---|---|---|
| | | Bukima | Nyambono | Buruma | Murangi |
| | | Nyamimange | Kyanyari | Tegeruka | Butuguri |
| | | Suguti | Bukumi | Mugango | Masaba |
| | | Muriaza | Bukabwa | Buswahili | Bwiregi |
| Pwani | Rufiji | Ngorongo | Kibiti | Bungu | Chumbi |
| | | Mchukwi | Ruaruke | Mahege | Ikwiriri |
| | | Maparoni | Mbwara | Mtunda | Salale |
| | | Mgomba | Mwaseni | Mbuchi | Kiongoroni |
| Rukwa | Sumbawanga Rural | Milepa | Kaengesa | Muze | Kasanga |
| | | Matai | Sandulula | Sopa | Mwimbi |
| | | Kalambazite | Mtowisa | Kaoze | Laela |
| | | Mpui | Lusaka | Mambwekenya | Miangalua |
| Replacement Wards | | | | | |
| **Region** | **District** | **Handwashing** | **Sanitation** | **Sanitation and HW** | **Control** |
| Iringa | Iringa Rural | Magulilwa | Mgama | Kihorogota | Itunundu |
| Kagera | Karagwe | Ihembe | Nyabiyonza | Nyakasimbi | Rugu |
| Mtwara | Masasi | Nanyumbu | Mchauru | Namatutwe | Chipuputa |
| | | Nanjota | Lulindi | Mbuyuni | Napacho |
| Dodoma | Mpwapwa | Mima | Mlunduzi | Berege | Luhundwa |
| | Kondoa | Chemba | Masange | | |
| | | Ovada | | | |
| Manyara | Kiteto | Songambele | Engusero | Njoro | Lengatei |
| Rukwa | Sumbawanga Rural | Katazi | Mwazye | Kipeta | Msanzi |
| | | | | | |

**Note: Districts highlighted in yellow are phase 1.**

APPENDIX D: Key Evaluation Design Features for Consideration of Implementing Firm

The following aspects of the Tanzania Handwashing and Sanitation Promotion impact evaluation design are of particular relevance for the design and implementation of the promotion activities.

1. There are two critical aspects relating to timing of the intervention
    a. Promotion activities should NOT start in an evaluation ward (190 of the 200 intervention wards) until after the baseline data collection is completed. 10 pilot wards have been selected where the promotion activities can initiate independent of the data collection (5 handwashing and 5 sanitation).
    b. By assigning districts to early and later intervention phases, the evaluation may be able to estimate the short-term effects of the regional mass media interventions. Currently, 5 districts have been assigned to phase 1, and 5 to phase 2. Depending on the mode of operations for the promotion, alternative designs can also be considered. However, the firm should consider the feasibility of scaling up at the district level, according to an agreed workplan with the evaluation team.
2. Local interventions must be confined to activities within a Ward, and not spill over into adjacent wards. Any activities that spill over Ward boundaries will be classified as regional interventions, and will not be captured as impacts under the primary evaluation design. Since the impact evaluation will focus on estimating the impact of the local interventions, it is critical that “powerful” interventions be implemented at the local level.
3. Wards have been assigned to one of three treatment groups. The handwashing intervention will target all wards within groups (T1) and (T3). The sanitation intervention will target all wards within groups (T2) and (T3). NO local interventions should be implemented in the comparison group C1. It is recommended that the implementing agencies assess the wards that have been selected for intervention, and advise the evaluation group if any logistical difficulties are anticipated with one or more wards. If a ward originally chosen for intervention “falls out” of the sample, the evaluation group should be consulted for an appropriate replacement.

**APPENDIX E: EVALUATION DESIGN LOG**

Instructions: Please document any changes to the Tanzania Handwashing and Sanitation impact evaluation design (as of December, 2007)

| | **Person/Date** | **Reason for Modification** | **Description of Modification** |
|---|---|---|---|
| 1. | | | |
| 2. | | | |
| 3. | | | |
| 4. | | | |
| 5. | | | |
| 6. | | | |
| 7. | | | |
| 8. | | | |
| 9. | | | |
| 10. | | | |
| 11. | | | |
